# Embryonic lineage-specific iPSC-derived mesenchymal stem/stromal cells exhibit different morphologies and intrinsic functions

**DOI:** 10.1016/j.isci.2025.114482

**Published:** 2025-12-18

**Authors:** Linh Nguyen, Souta Motoike, Denise Zujur, Keiko Yoshizawa, Yasuhiro Takashima, Akiyoshi Uezumi, Kazuhiro Furuhashi, Shoichi Maruyama, Yonghui Jin, Junya Toguchida, Hidetoshi Sakurai, Makoto Ikeya

**Affiliations:** 1 Department of Clinical Application, Center for iPS Cell Research and Application (CiRA), Kyoto University, Kyoto, Japan; 2 Department of life Science Frontiers, Center for iPS Cell Research and Application (CiRA), Kyoto University, Kyoto, Japan; 3 Division of Cell Heterogeneity, Medical Research Center for High Depth Omics, Medical Institute of Bioregulation, Kyushu University, Fukuoka, Japan; 4 Department of Nephrology, Nagoya University Graduate School of Medicine, Nagoya, Japan; 5 Department of Regeneration Sciences and Engineering, Kyoto University, Kyoto, Japan; 6 Department of Fundamental Cell Technology, Center for iPS Cell Research and Application, Kyoto University, Kyoto, Japan

**Keywords:** Cell biology, Developmental biology

## Abstract

Mesenchymal stem/stromal cells (MSCs) have great potential in regenerative medicine owing to their multilineage differentiation capacity. However, tissue-derived MSCs (tMSCs) exhibit inconsistent characteristics. Although induced pluripotent stem cell (iPSC)-derived MSCs (iMSCs) are a potential solution, the effect of different embryonic lineages on their properties remains unknown. We generated MSCs from human iPSCs via five lineage-specific routes: cranial neural crest, trunk neural crest, paraxial mesoderm (somite), lateral plate mesoderm, and limb mesenchyme. All types met established MSC criteria yet differed in morphology, proliferation, and differentiation capacity. Somite-, cranial neural crest-, and limb mesenchyme-derived MSCs showed higher osteogenic potential, whereas somite-derived MSCs also showed high chondrogenic potential but were prone to hypertrophy. Limb mesenchyme-derived MSCs showed the highest adipogenic potential. Transcriptomic profiles indicated distinct clusters within iMSCs. Despite variances, a high correlation level existed between iMSCs and tMSCs. Therefore, iMSCs are potential alternatives to tMSCs in regenerative medicine.

## Highlights

• iMSCs were differentiated from the same iPSC line via 5 different embryonic origins
• Different iMSC types displayed different intrinsic characteristics
• Somite-derived iMSCs (SM-iMSCs) showed higher osteogenic differentiation potential
• Cranial neural crest-derived iMSCs (cNCC-iMSCs) have more chondrogenic potential

## Graphical abstract

**Figure:**
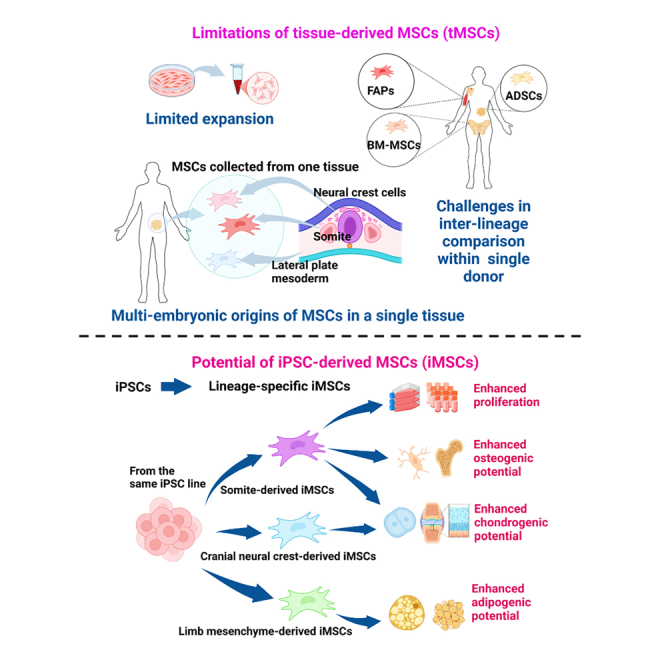

## Introduction

Over the past 30 years, mesenchymal stem/stromal cells (MSCs) have been extensively studied for their applications in regenerative medicine.^1^ These heterogeneous^2^^,^^3^ cells have been widely studied for their differentiation potential, immunoregulatory functions, and exosome secretion.^4^^,^^5^^,^^6^^,^^7^ However, tissue-derived (or primary) MSCs (tMSCs) exhibit a restricted expansion capacity and progressive decline in multiple differentiation capacities during prolonged culture.^8^^,^^9^ These factors may compromise their long-term effectiveness in regenerative medicine. Furthermore, MSCs derived from different tissues showed different characteristics and may be optimal for different clinical applications.^10^ However, comparing MSCs derived from multiple tissues of the same donor is particularly challenging because of practical and biological constraints, such as tissue and donor variabilities.^11^ Additionally, MSCs derived from a single tissue can originate from multiple embryonic lineages, including the paraxial mesoderm, lateral plate mesoderm (LPM), and neural crest cells.^12^^,^^13^^,^^14^^,^^15^^,^^16^ This intrinsic heterogeneity complicates efforts to study MSC biology and optimize their therapeutic applications, along with purification of single origin-derived MSCs collected from one tissue.

Induced pluripotent stem cells (iPSCs) are created by reprogramming somatic cells to an epiblast-like pluripotent state, enabling their unlimited expansion and granting them vast capacities to differentiate into various cell types, contributing to the advancement of our understanding of a wide range of diseases as well as the development of many cell therapy applications.^17^^,^^18^^,^^19^^,^^20^^,^^21^^,^^22^ Several protocols have reported the induction of MSCs from human iPSCs^23^^,^^24^^,^^25^^,^^26^^,^^27^^,^^28^ promising a generation of iPSC-derived MSCs (iMSCs) that can overcome the limitations of tMSCs.^11^ With their capacity for unlimited expansion derived from a single embryonic lineage, iMSCs address the issues of restricted proliferation and lineage diversity observed in tMSCs.

While current protocols to derive MSCs from iPSCs are effective, the MSCs thus generated often lack thorough characterization or the induction process does not follow lineage-specific developmental pathways; moreover, the generated iMSCs do not always retain the properties of the tMSCs.^24^ Therefore, the comprehensive characterization of iMSCs derived from different developmental pathways is an area of great interest. Detailed characterization is essential to fully harness the therapeutic potential of iMSCs in regenerative medicine and disease modeling as well as for the utilization of MSCs derived from various tissues.^1^^,^^11^ Therefore, we focused our efforts on deriving iMSCs from various tissues and thoroughly characterizing them based on International Society for Cellular Therapy (ISCT)’s minimum criteria of MSCs.^18^^,^^19^

In this study, we differentiated five different types of iMSCs using previously reported protocols, with some modifications. We then compare these 5 different types of iMSCs in terms of their morphology, proliferation, and differentiation capacities.

## Results

### iMSC differentiation via different embryonic intermediates

**Figure 1:**
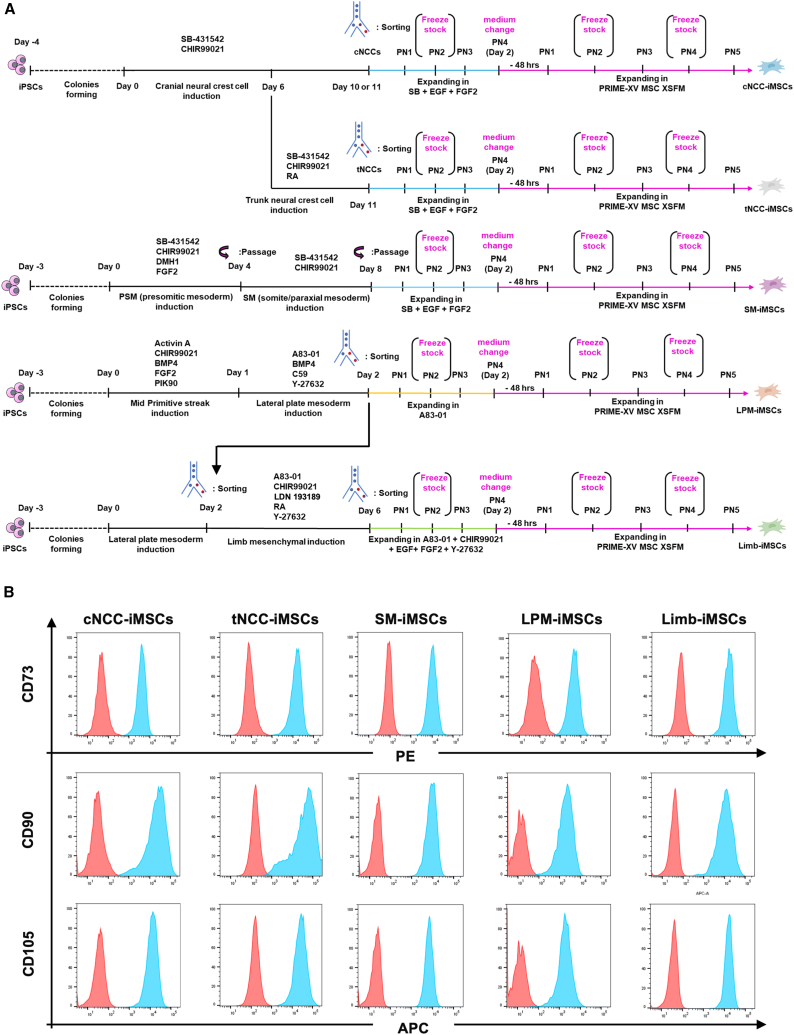
MSC Generation from different developmental origins (A) Protocols for deriving iMSCs from iPSCs involve differentiating iPSCs into various intermediate stages, followed by expansion and induction into iMSCs in xeno-free environment. (B) Successful differentiation of iMSCs demonstrated through FACS analysis based on positive expression of MSCs’ defined markers CD73, CD90, and CD105. The red histogram represents isotype controls, and the blue histogram shows samples stained with antibodies.

We recently reported an efficient protocol for inducing iMSCs from human iPSCs via an intermediate stage of neural crest cells.^25^^,^^26^ Neural crest cells emerge as neural plate folds in the early stages of neurulation. To generate cranial neural crest (cNCC)-derived iMSCs (cNCC-iMSCs), we precisely modulated the signaling pathways involved in neural crest cell induction (Figure 1A). Initially, iPSCs were cultured for 4 days in iPSC maintenance medium (AK03N) to allow colony formation and achieve the desired confluence (Figure S1A). On day 0 (the day when induction started), the Wnt signaling pathway was activated and the transforming growth factor (TGF)-β signaling pathway was inactivated by supplementing the culture medium (StemFit Basic 03) with 1 μM CHIR99021 and 10 μM SB431542 to direct the cells toward neural crest differentiation. Cells were then harvested and sorted based on the expression of CD271, a neural crest cell marker, on day 10 or 11. The efficiency of this protocol was validated by the high yield of CD271-positive cells (>80%) (Figure S1B). For trunk neural crest cell (tNCC) differentiation, retinoic acid (RA), which is crucial during later differentiation,^21^^,^^22^ was added to the culture medium on day 6 and cells were harvested and sorted based on the expression of CD271. The efficiency of the induction of neural crest cells was confirmed by fluorescence-activated cell sorting (FACS) analysis. Both cNCCs and tNCCs expressed the definitive neural crest marker *SOX10* (Figure S1C). Immunostaining also showed that neural crest cells expressed specific markers of replated sorted cells, such as SOX10*,* p75NTR (or CD271), and TWIST1 (Figure S1D). Specific posterior markers, such as *HOXB2*, *HOXB5,* and *HOXB8*,^21^^,^^22^^,^^27^^,^^28^^,^^29^^,^^30^^,^^31^^,^^32^ are significantly upregulated in tNCCs, but not in cNCC cells (Figure S1E). Global gene expression analysis using a heatmap revealed the upregulation of established tNCC markers, such as HOXB6 and PHOX2B,^33^^,^^34^^,^^35^^,^^36^ in tNCCs. tNCCs originated from the posterior region of the neural tube, caudal to the hindbrain, and do not express markers from hindbrain-derived (cranial/vagal) and sacral NCCs.^28^^,^^29^^,^^30^^,^^31^ Consistently, our data showed minimal expression of markers typically associated with hindbrain and sacral neural crest lineages (Figure S2A). These findings were corroborated by qPCR results, which confirmed the upregulation of *CDX2* and *PHOX2B* (Figure S2B). The volcano plot showed that the upregulation of differentiated genes, including *HOXB5*, *HOXB6*, and *HOXB8*, in tNCCs aligns with the heatmap and qPCR results (Figure S3A). Further, Gene Ontology (GO) term analysis revealed that both cNCC and tNCC samples were enriched in NCC development and differentiation pathways (Figures S3B and S3C).

To obtain paraxial mesoderm from iPSCs, we inhibited TGF-β and bone morphogenetic proteins (BMP) signaling pathways using SB431542 and DMH1 (dorsomorphin homolog 1), respectively, while promoting Wnt and fibroblast growth factor (FGF) signaling pathways using CHIR99021 and FGF2, respectively, to achieve the presomitic mesoderm stage (Figures 1A and S4A), as described by Nakajima et al.^37^ By day 4, more than 99% of the cells expressed the paraxial mesoderm marker *DLL1* (Figure S4B). Subsequently, Wnt signal and TGF-β inhibition signal were maintained until day 8. At this point, the somitic markers *PAX3* and *MEOX1* were assessed through mRNA expression analysis, revealing significant upregulation (Figure S4C).

Several studies have reported the induction of the LPM and the identification of specific markers for this population. We adapted the protocol described by Loh et al.^38^ with some modifications (Figure 1A). After colony formation for 3 days, activin A, CHIR99021, BMP4, FGF2, and PIK90 were treated for 1 day and A83-01, BMP4, C59, and Y-27632 were treated for another 1 day. By day 2, a substantial proportion of cells expressed KDR (or CD309) (Figure S5A), a specific marker of the LPM.^39^^,^^40^ Other defined markers of the LPM, such as *FOXF1*, *HAND2* and *ISL1*, were also significantly upregulated (Figure S5B). The PDGFRA^+^ KDR^+^ population was sorted for further differentiation and to ensure the exclusion of undifferentiated iPSCs. This dual marker expression is also essential for identifying and characterizing cardiac progenitor-derived LPM.^39^^,^^40^

To induce limb mesenchyme progenitors from the sorted LPM population, we adapted the protocol developed by Yamada et al.,^41^ with certain modifications (Figure 1A). From days 0 to 2, LPM was induced and sorted as described above. Then on day 2, limb mesenchyme induction started by culturing sorted LPM in A83-01-, CHIR99021-, LDN193189-, RA-, and Y27632-containing medium. The primary modification involved the addition of RA during the induction process (Figure S5C), as RA signaling is crucial for limb development and has been demonstrated to be essential for the proper formation of limb mesenchyme (Figure S5D).^42^^,^^43^^,^^44^ On day 6, the efficiency of limb mesenchyme induction was assessed using FACS, followed by sorting of cells positive for limb mesenchyme markers, specifically *PDGFRA*^*+*^ *and KDR*^*-*^ (Figure S6A). Additionally, other limb mesenchyme markers, including *PITX1*, *IRX3*, and *TBX5*, were significantly upregulated (Figure S6B). RNA sequencing (RNA-seq) further confirmed the upregulation of key signaling molecules essential for limb development, such as *WNT* and *HOXB* genes,^41^^,^^45^ in the sorted cells collected on day 6 (Figure S6C). These successful protocols for inducing intermediates such as NCCs, somites (SM), LPM, and limb progenitors were also applied to another iPSC line (1383D6), resulting in highly efficient generation of comparable intermediates (Figure S7).

Following induction and purification of the intermediates, the cells were expanded in an appropriate growth medium (Figure 1A). NCCs and SM cells were expanded in a medium specifically designed for NCCs, which includes a TGF-β inhibitory signal, along with epidermal growth factor (EGF) and FGF2 signals.^25^^,^^46^ Owing to the upregulation of the cardiac marker *NKX2-5* in the presence of EGF and FGF2 signals (Figure S6D), the LPM was expanded in a medium devoid these two signals, resulting in an increase in cell numbers over time (Figure S6E), suggesting the feasibility of expanding these cells in this modified medium. Limb mesenchyme progenitors were expanded in a medium supplemented with Wnt signaling molecules, which is crucial for the expansion of the limb mesenchyme, as demonstrated by Yamada et al. and Geetha-Loganathan et al.^41^^,^^45^ The intermediates were cultured in the expansion medium until passage number four (PN4); thereafter, the medium was switched to a commercially available xeno-free medium (PRIME-XV Expansion medium XSFM) that supports the expansion and transition of these cells into MSCs (Figure 1A). While the exact composition is not publicly disclosed, its consistent use in multiple published protocols promotes mesenchymal characteristics *in vitro* and the expansion of tMSCs.^25^^,^^47^^,^^48^ After five passages of expansion, the cells were analyzed for the expression of MSCs’ recognized surface markers—CD73, CD90, and CD105. All the induced iMSC types demonstrated high expression levels of these markers, with over 90% of the cells showing strong positivity (Figure 1B). In addition, the cells did not express the hematopoietic markers, CD34 and CD45, which are negative markers of MSCs (Figure S8). For consistency, all comparative assays were performed at PN5, selected on the basis of pilot passage tracking (PN3–PN6), in which cNCC-, SM-, and Limb-iMSCs showed stable MSC-marker profiles across this window, while LPM-iMSCs reached their highest marker expression at PN5–PN6; PN5 was, therefore, chosen to minimize passage-related variability while ensuring adequate yield.

### MSCs derived from iPSCs via different embryonic lineages exhibit distinctive morphology, proliferation rates, and immunoregulatory potential

**Figure 2:**
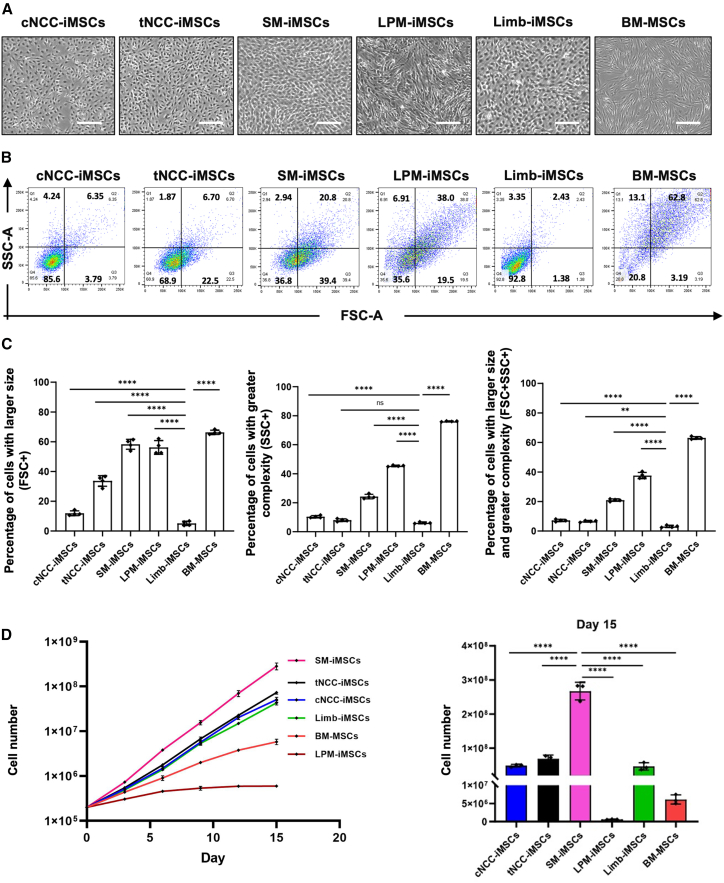
MSCs from different developmental origins display variances in terms of morphology and proliferation (A) Phase-contrast images showed variances in MSCs’ morphology as LPM-iMSCs have elongated, spindle shape, while other iMSCs are smaller in size (scale bars, 200 μm). (B) Cell size and granularity were measured by forward scatter and side scatter (representative data, 3 independent experiment). (C) Bar graph shows the percentage of cells with larger size and more complexity in each iMSC types with BM-MSCs as control. (Data are presented as mean ± SD, *n* = 4 independent experiments, one-way ANOVA followed by Tukey’s test). (D) Line graph shows different proliferation rate among different types of iMSCs, as SM-iMSCs have the highest proliferation rate and LPM-iMSCs have the lowest proliferation rate (*x* axis as log10 scale), and the bar graph shows SM-iMSCs have the highest number of cells yielded while LPM-iMSCs have the lowest. (*n* = 3, independent experiments.) Data are presented as mean ± SD, *n* = 3, independent experiments, one-way ANOVA followed by Tukey’s test, full results of all pairwise comparisons are provided in Table S2. *p* value notification: ∗*p* < 0.05; ∗∗*p* < 0.01; ∗∗∗*p* < 0.001, ∗∗∗∗p < 0.0001.

After inducing differentiation in various iMSC populations, we conducted a comprehensive analysis of their morphology, proliferation, and functional capacity to identify potential differences. Phase-contrast images of cells at passage 5 revealed that while all iMSCs generally exhibited a small, spindle-shaped morphology, notable variations were observed among different types (Figure 2A). Specifically, MSCs derived from the lateral plate mesoderm (LPM-MSCs) displayed a more heterogeneous population, with cells exhibiting a larger and more elongated morphology than other iMSCs. This morphological diversity was also observed in bone marrow-derived MSCs (BM-MSCs), which were cultured under identical conditions with the iMSCs, as both elongated spindle-shaped and smaller cells. These differences in terms of morphology have also been observed in lineage-specific iMSC types derived from iPSC 1383D6 (Figure S9). Following the initial morphological analysis, we conducted flow cytometry to quantitatively assess the size and internal complexity of the iMSCs using forward scatter (FSC) and side scatter (SSC) parameters. FSC indicates cell size based on the amount of laser light diffracted around the cell, and SSC provides information on the internal complexity of cells by measuring light scattered by intracellular structures.^49^^,^^50^^,^^51^ Consistent with the microscopic observation, LPM-MSCs exhibited the highest proportion of cells of large size (high FSC signal) and increased internal complexity (high SSC signal) (Figures 2B and 2C). Notably, despite being derived from the LPM, iMSCs derived from limb mesenchyme (Limb-iMSCs)—a derivative of the lateral mesoderm—generally consisted of smaller cells with lower granularity. Specifically, only 3.1% ± 0.75% of Limb-iMSCs were larger cells that showed high FSC intensity and only 6.11% ± 0.57% displayed elevated internal complexity (Figure 2C). Additionally, both cNCCs and tNCC-derived iMSCs (tNCC-iMSCs) exhibited smaller cell sizes and lower complexities, whereas approximately 50% and 30% of SM-derived iMSCs (SM-iMSCs) exhibited larger size and greater complexity, respectively.

Next, we assessed the proliferation rate of iMSCs using BM-MSCs as controls. All MSCs were cultured in identical expansion media, seeded at the same cell density for each passage, and passaged for the same number of times for the same duration. Somite-derived MSCs (SM-MSCs) demonstrated the highest proliferation rate, resulting in the highest number of cells after 15 days (Figure 2D). This was followed by cNCC-iMSCs, tNCC-iMSCs, and Limb-iMSCs. In contrast, BM-MSCs exhibited a moderate proliferation rate and LPM-iMSCs yielded the lowest number of cells (Figure 2D). In summary, iMSCs generally exhibit a higher proliferation rate than BM-MSCs, consistent with several reports.^8^^,^^52^^,^^53^

Following the assessment of proliferation rates, we next investigated the immunoregulatory response of the iMSCs in comparison to that of BM-MSCs using interferon gamma (IFN-γ) treatment to simulate an inflammatory environment. Briefly, when MSCs reached confluence at PN5, they were treated with IFN-γ for 24 h, after which the expression levels of key immunomodulatory markers, *PD-L1* and *IDO1*, were evaluated (Figure S10A).^25^^,^^29^^,^^54^ *IDO1* and *PD-L1* were chosen for the preliminary assay since they yield early, robust, and easy-to-standardize monoculture assays, whereas *IL-10* and *TGF-β* often require co-culture or dual licensing and, therefore, were reserved for follow-up.^55^^,^^56^ Figure S10B demonstrates a significant upregulation of *IDO1* in both iMSCs and BM-MSCs following 24 h of IFN-γ treatment, highlighting the immunoregulatory potential across all iMSC types. Similarly, *PD-L1* expression was markedly increased in all iMSCs, further supporting its role in immune modulation. However, the varying expression levels of these markers among iMSCs suggest differences in their immunoregulatory capacities. Notably, SM-iMSCs exhibited the lowest expression of both markers, whereas LPM-iMSCs showed the highest *PD-L1* expression after IFN-γ treatment. Of note, LPM-iMSCs exhibited baseline *PD-L1* expression even without IFN-γ stimulation. This suggests that LPM-iMSCs may be suitable for therapeutic applications requiring immunosuppression; however, more functional validations are required, and although LPM-iMSCs proliferate slowly, this limitation can be mitigated by prolonging expansion at the LPM-intermediate stage, increasing initial seeding density, and leveraging LPM-iMSC-derived exosomes.

### iMSCs from different embryonic lineages display different osteogenic and adipogenic capacities

**Figure 3:**
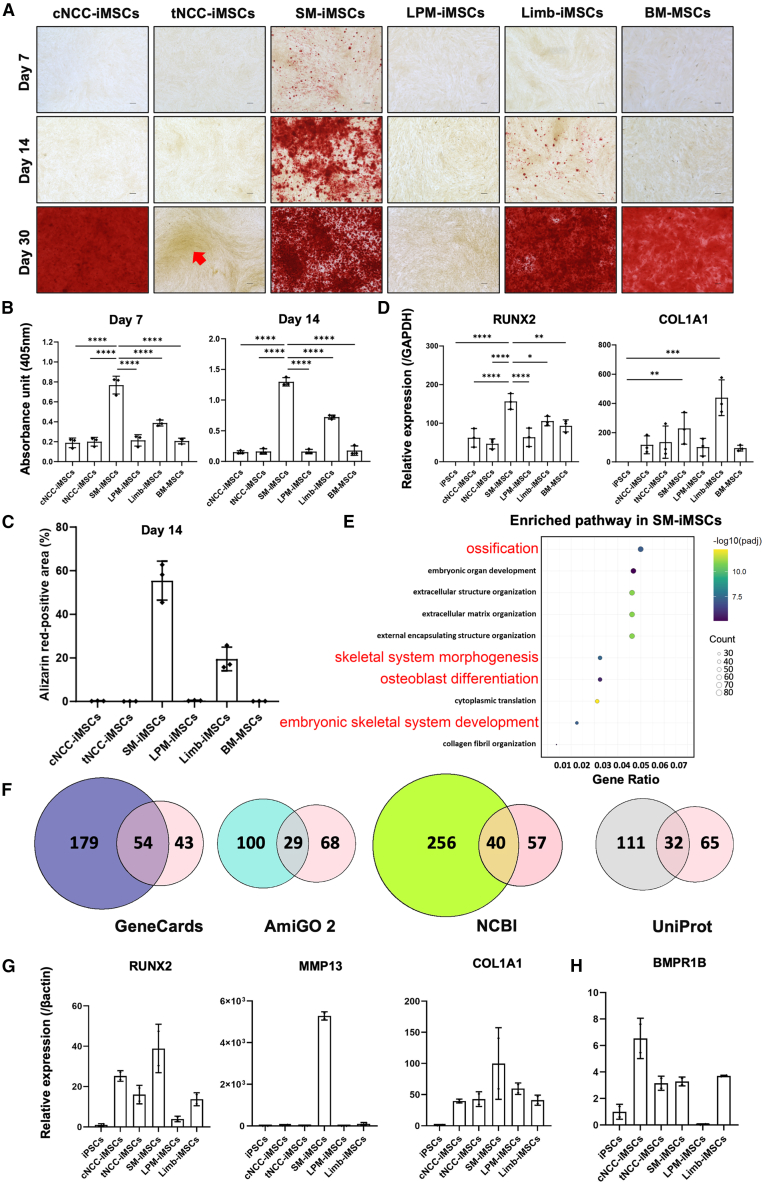
Osteogenic differentiation potential varies among MSCs derived from different developmental origins (A) Bright-field microscopy images reveal that SM-iMSCs undergo early calcification detectable by alizarin red staining on day 7, with Limb-iMSCs showing calcification by day 14, followed by cNCC-iMSCs, while tNCC-MSCs show appearance of high-density cell nodule (red arrow) and LPM-iMSCs are only in the early stage of mineralization. (B) Quantification of calcification by alizarin red staining at days 7 and 14 shows the highest calcification levels in SM-iMSCs, followed by Limb-iMSCs. (Data are presented as mean ± SD, *n* = 3, independent experiments, one-way ANOVA followed by Tukey’s test, full results of all pairwise comparisons are provided in Table S2). (C) Alizarin red-staining areas of SM-iMSCs were highest among the iMSCs after 14 days in osteogenic culture. (Data are presented as mean ± SD, *n* = 3, independent experiments). (D) Comparative qPCR analysis at day 7 shows that all iMSC types, including BM-MSCs, significantly upregulate *RUNX2* gene, a key regulator of osteogenesis, with SM-iMSCs displaying the highest level of *RUNX2* expression. (Data are presented as mean ± SD, *n* = 3, independent experiments). (E) Enriched pathways in SM-iMSCs related to ossification (*n* = 2, independent experiments). (F) Identified ossification pathway in GO terms analysis validated by comparing extracted genes with traditional databases such as GeneCards, UniProt, AmiGo 2, and NCBI. (G) qPCR result validates the RNA-seq analysis as osteogenesis-related genes such as *RUNX2, MMP13*, and *COL1A1* are upregulated in SM-iMSCs. (Data are presented as mean ± SD, *n* = 2, independent experiments). (H) Osteogenic marker *BMPR1B* upregulated in all iMSCs except for LPM-iMSCs, consistent with the functional experiment. (Data are presented as mean ± SD, *n* = 2, independent experiments). *p* value notification: ∗*p* < 0.05; ∗∗*p* < 0.01; ∗∗∗*p* < 0.001; ∗∗∗∗*p* < 0.0001).

After comparing iMSCs based on their morphology, proliferation, and immunoregulatory potential, we assessed their differentiation potential, particularly osteogenesis, a hallmark characteristic of MSCs. Using BM-MSCs as a positive control, we seeded equal numbers of cells and initiated osteogenic induction on day 3. Alizarin red S staining, a widely accepted method, was used to detect calcium deposition *in vitro*; it identifies mineralization relevant to osteogenesis—including both calcium phosphate and calcium hydroxyapatite in bone^57^^,^^58^ in samples. The results showed that SM-iMSCs were the first to exhibit calcification (the accumulation of calcium salts, predominantly calcium phosphate, the main inorganic component of bone matrix, together with calcium hydroxyapatite) detected by positive alizarin red staining on day 7. Limb-iMSCs and cNCC-iMSCs were the next to exhibit calcification on days 14 and 30, respectively (Figure 3A). In contrast, tNCC-iMSCs displayed lower osteogenic potential with modest staining of focal high-density cell nodules (red arrow, Figure 3A), while LPM-iMSCs showed minimal to no staining. This suggests an alignment between lineage priming iMSCs and the tissue of origin, as SMs form the axial skeleton, NCCs contribute to cranial bones, and the limb mesenchyme forms limb bones.^59^ Alizarin red staining indicated that SM-iMSCs exhibited the highest level of calcification on days 7 and 14 (Figure 3B), and analysis of the alizarin red-stained area using ImageJ showed the largest positive area in SM-iMSCs, followed by Limb-iMSCs at day 14 (Figure 3C). Moreover, osteogenic markers, such as *RUNX2* and *COL1A1,* examined on day 7 as the time when earliest osteogenesis was detected in samples, were upregulated in all iMSCs (Figure 3D), indicating enhanced osteogenic differentiation. Given the superior osteogenic potential of SM-iMSCs and the lower capacity of LPM-iMSCs, we conducted GO term enrichment analysis of the differentially expressed genes at the iMSC stage to investigate the molecular mechanisms underlying osteogenesis in iMSCs. The analysis revealed enrichment of pathways related to ossification and skeletal morphogenesis in SM-iMSCs (Figure 3E), as well as in Limb-iMSCs and cNCC-iMSCs (Figures S11A and S11B), which is consistent with the results of alizarin red staining and osteogenic gene expression after induction. Subsequently, we compared ossification-related genes from the GO terms analysis with common databases such as GeneCards, AmiGO 2, NCBI, and UniProt to validate the analysis (Figure 3F). Further, qPCR analysis also confirmed the upregulation of osteogenic markers, including *RUNX2*, *MMP13*, and *COL1A1*, in SM-iMSCs among all iMSCs (Figure 3G) in the same samples used for RNA-seq, thereby validating these results. The osteogenesis-related marker *BMPR1B* was upregulated in all iMSCs, except for LPM-iMSCs, which was consistent with the results of the functional analysis (Figure 3H). The qPCR results suggest stage-specific osteogenesis: *RUNX2* and *MMP13* mark early commitment, whereas *COL1A1* mRNA indicates active-matrix synthesis. By day 7, SM-iMSCs appear past the peak of collagen synthesis, resulting in *COL1A1* slight downshift with mineralization evident, while Limb-iMSCs show higher *COL1A1*, yet limited, positive alizarin red-stained area, consistent with ongoing matrix build-up and mineralization at day 14. Similar pattern in osteogenesis of 1383D6-derived iMSCs has been observed, as SM-iMSCs displayed the highest potential, followed by Limb-iMSCs and cNCC-iMSCs (Figure S12). These findings emphasize the importance of embryonic origin in defining the osteogenic potential of iMSCs and suggest that SM-iMSCs could be particularly useful for bone regeneration therapies, warranting further exploration of their clinical application.

**Figure 4:**
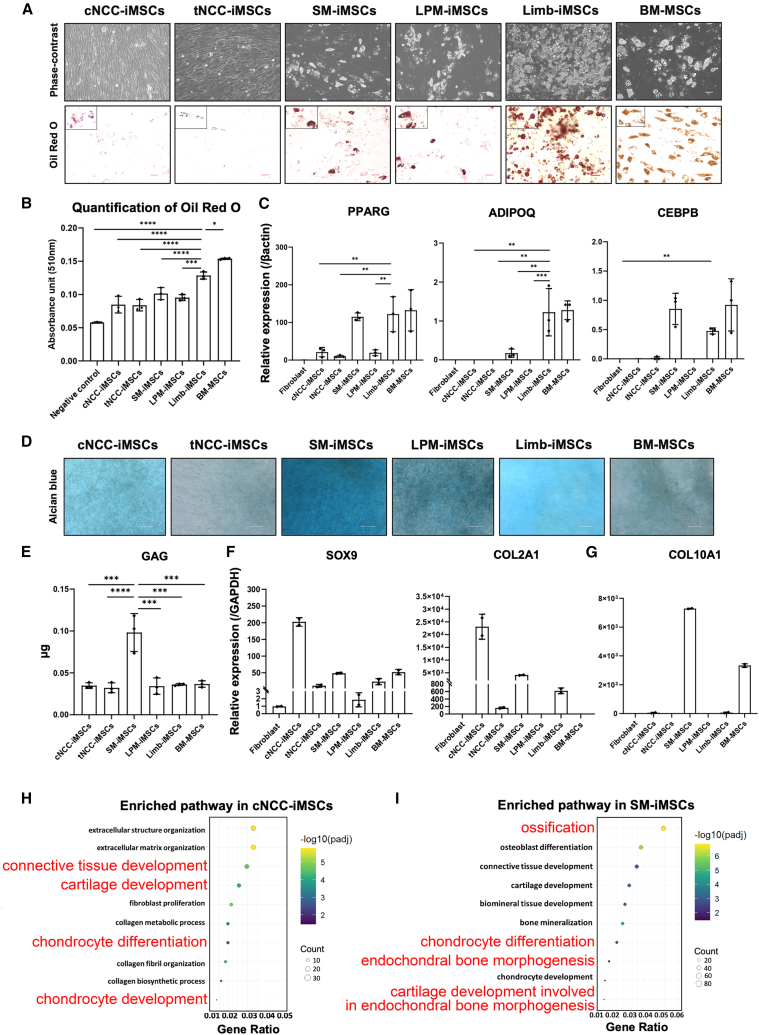
iMSCs display different adipogenic and chondrogenic differentiation potential (A) Phase-contrast images and oil red O staining showed that cells with accumulated triglyceride droplets appeared in all samples and were highest in number in the Limb-iMSC samples (scale bars, 50 μm, representative data from 3 independent experiments). (B) Quantitative analysis of oil red O staining showed that Limb-iMSC samples exhibited the highest results among all samples. (Data are presented as mean ± SD, *n* = 3, independent experiments). (C) qPCR at D28 showed *PPARG* upregulation in all samples, with *ADIPOQ* and *CEBPB* significantly higher in Limb-iMSCs. (Data are presented as mean ± SD, *n* = 3, independent experiments). (D) Alcian blue staining demonstrated that all iMSC types have chondrogenic potential, as evidenced by the deposition of glycosaminoglycans in the extracellular matrix after 7 days of culture in chondrogenic differentiation medium (scale bars, 200 μm, representative data from 3 independent experiments). (E) Quantitative analysis of extracted GAGs showed that the SM-iMSC samples had the highest GAG content among all groups; “μg” denotes the total mass of sulfated glycosaminoglycans (sGAGs) quantified per iMSC pellet. (Data are presented as mean ± SD, *n* = 3, independent experiments, one-way ANOVA followed by Tukey’s test, full results of all pairwise comparisons are provided in Table S2, *p* value notification: ∗*p* < 0.05; ∗∗*p* < 0.01; ∗∗∗*p* < 0.001; ∗∗∗∗*p <* 0.0001). (F) qPCR results indicated that the chondrogenic marker *SOX9* was upregulated in all iMSC samples compared to negative control fibroblasts, confirming successful chondrogenic induction, with *COL2A1* expression significantly higher in the cNCC-iMSC samples. (n = 2, independent experiments). (G) Specific hypertrophic marker *COL10A1* significantly upregulated in SM-iMSC samples. (n = 2, independent experiments). (H) Further GO term analysis reveals pathways related to chondrogenesis in cNCC-iMSCs compared to LPM-iMSCs. (n = 2, independent experiments). (I) Ossification and hypertrophy-related pathways enriched in SM-iMSCs (n = 2, independent experiments).

Next, we investigated the adipogenic differentiation potential of iMSCs of various origins by combining morphological assessments, lipid staining techniques, and quantitative mRNA expression analysis, using BM-MSCs as a positive control. The adipogenic induction medium used in this study did not contain exogenous lipids, ensuring that lipid accumulation reflects intracellular synthesis rather than uptaking lipids from the culture environment. After 28 days of induction, phase-contrast microscopy coupled with oil red O staining allowed visualization of the accumulation of intracellular triglyceride droplets, a hallmark of adipogenic differentiation (Figure 4A). The staining results showed that all iMSC samples at day 28 developed cells containing lipid droplets, indicating successful differentiation across all groups. Notably, Limb-iMSCs appeared to have a greater number of cells with lipid droplets than other iMSCs. To objectively quantify lipid accumulation across samples, we measured the amount of oil red O stain extracted from the cells. The quantitative data confirmed that the Limb-iMSC samples exhibited the highest absorbance (Figure 4B), consistent with the morphological observations. At the molecular level, we conducted qPCR to measure the expression levels of the key adipogenic marker *PPARG* after induction and confirmed its higher expression in Limb-iMSC samples (Figure 4C). Furthermore, Limb-iMSCs and SM-iMSCs showed higher expression of *ADIPOQ* and *CEBPB* than other iMSCs after induction (Figure 4C), suggesting greater adipogenic potential in these iMSCs types. Further, transcriptome analysis of significantly upregulated genes in Limb-iMSCs compared with established databases, such as KEGG and Reactome, revealed enrichment in adipogenesis-related pathways, including lipid, glycerophospholipid, and fatty acid metabolism (Figure S13), consistent with the results of functional and qPCR analyses.

### Differential chondrogenic potential of iMSCs in 2D and 3D cultures

To evaluate the differential chondrogenic potential of iMSCs of various embryonic origins, we initially used Alcian blue staining, a histological technique that specifically binds to glycosaminoglycans (GAGs) in the extracellular matrix, which are key components of cartilage.^60^^,^^61^ After two-dimensional (2D) chondrogenic differentiation, staining on day 7 demonstrated that all iMSC types had chondrogenic potential, as evidenced by the deposition of GAGs (Figures 4D and S14). To quantitatively compare the chondrogenic capacity of each iMSC type, we extracted and quantified GAGs from the cultures. SM-iMSC samples exhibited the highest GAGs content among all groups (Figure 4E), indicating that SM-iMSCs have an increased ability to produce cartilage-specific extracellular matrix components, which could be attributed to their rate of proliferation, which was the highest among all the studied cell types (Figure 2D).

Further molecular characterization was performed using qPCR to assess the expression levels of key chondrogenic markers. After induction, there was significant upregulation of *SOX9* in all iMSC samples compared with that in negative control fibroblasts, confirming successful chondrogenic induction across the board (Figure 4F). Notably, the expression of *COL2A1*, which encodes type II collagen, a major structural protein in cartilage,^62^ was significantly higher in the cNCC-iMSC samples (Figure 4F). This suggests that cNCC-iMSCs not only initiate chondrogenesis but also progress toward producing a mature cartilage matrix more effectively than other iMSC types. Furthermore, 7 days after chondrogenic induction, iMSCs were analyzed for the expression levels of the hypertrophic marker *COL10A1*, which serves as an indicator of calcified chondrogenic tissue.^62^ We observed that *COL10A1* expression was significantly upregulated in SM-iMSCs than in other iMSC types (Figure 4G). Further RNA-seq data analysis using GO terms in the iMSC stage (iMSCs of PN5) revealed the enrichment of chondrogenesis-related pathways in both cNCC-iMSCs and SM-iMSCs, whereas hypertrophy-related pathways were enriched only in SM-iMSCs (Figures 4H and 4I), consistent with the qPCR results. This finding provides insights into the selection of iMSCs for chondrogenic applications, particularly when the objective is to produce stable chondrogenic tissue.

**Figure 5:**
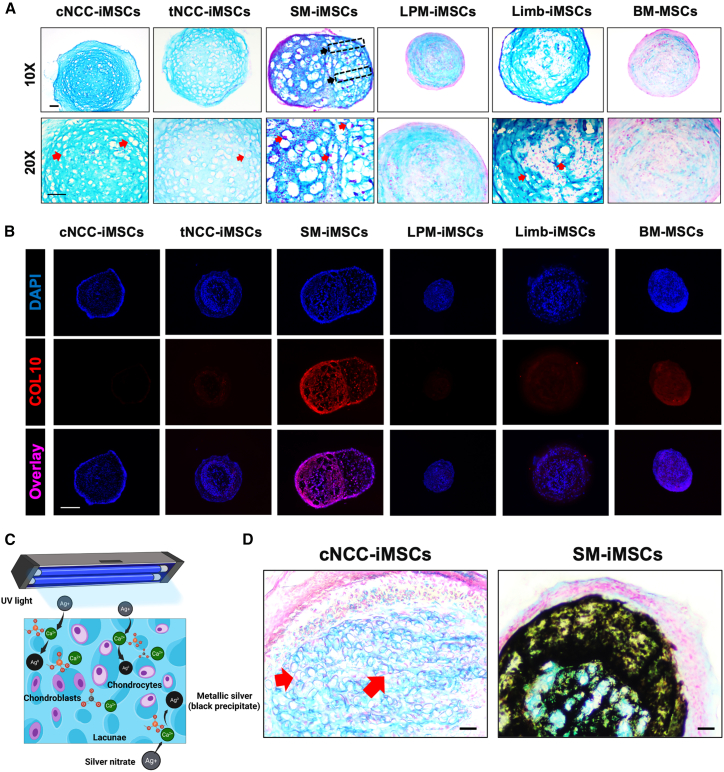
All iMSCs can be differentiated into chondrogenic spheroids in 3D culture (A) After 21 days of chondrogenic differentiation, all iMSC spheroids were stained with Alcian blue, indicating glycosaminoglycan production (scale bars, 50 μm, representative data from 3 independent experiments) with chondrocytes resting in well-structured lacunae (red arrows) and column-like structure in hypertrophic region in SM-iMSCs’ chondrogenic spheroid (black arrow). (B) Hypertrophic markers were strongly expressed in SM-iMSC chondrogenic spheroids and other iMSC samples, except for cNCC-iMSC chondrogenic spheroids (scale bar, 200 μm, representative data from 3 independent experiments). (C) Illustration explains the mechanism of von Kossa testing for dystrophic hypertrophy in chondrogenic pellet. (D) Images show that spheroids from cNCC-iMSCs were stable in the *in vivo* setting with chondrocytes resting in well-structured lacunae (red arrows), while spheroids from SM-iMSCs showed calcification by von Kossa test (scale bars, 50 μm).

Recognizing that three-dimensional (3D) culture systems more closely mimic the physiological conditions within the human body,^63^^,^^64^^,^^65^ we examined the chondrogenic differentiation of iMSCs in 3D spheroid cultures (Figure S15A). After 21 days of chondrogenic induction using an established 3D culture protocol^66^ with BM-MSCs serving as controls, chondrogenic spheroids were collected, sectioned, and stained with Alcian blue. All spheroids exhibited Alcian blue staining, confirming that all iMSC types differentiated into GAG-producing chondrocytes and expressed *COL2A1*, a specific chondrogenic marker (Figures 5A and S16). Phase-contrast images revealed noticeable differences in spheroid size among the iMSCs (Figures S15B and S15C), with the largest spheroids derived from SM-iMSCs and the smallest from LPM-iMSCs. Furthermore, spheroids from cNCC-iMSCs display well-formed lacunae, which are small cavities within the cartilage matrix housing chondrocytes.^67^^,^^68^ The presence of lacunae is characteristic of healthy cartilage tissue, indicating proper matrix deposition and organization.^68^ Chondrocytes were detected lying in well-structured lacunae in chondrogenic spheroids of cNCC-iMSCs, tNCC-iMSCs, SM-iMSCs, and Limb-iMSCs (Figure 5A, red arrows). In contrast, spheroids derived from LPM-iMSCs showed less-pronounced lacunae and irregular matrix organization, suggesting a less-mature chondrogenic phenotype or a different type of cartilage tissue. Additionally, hypertrophic-like regions with increased cell size and column-like structures were more prominent in spheroids derived from SM-iMSCs (Figure 5A, black arrows), indicating a tendency toward chondrocyte hypertrophy and potential progression to ossification.

To further investigate this hypertrophic tendency, we performed immunohistochemical analysis for COL10A1 in spheroids. Positive staining for COL10A1 was observed in all samples, except those derived from cNCC-iMSCs (Figure 5B). The absence of COL10A1 expression in cNCC-iMSCs suggested that these cells maintained a stable chondrogenic phenotype without advancing to hypertrophy. In contrast, the high expression of COL10A1 in SM-iMSCs (Figure 5B) indicates that although these cells have a high chondrogenic capacity, they may be predisposed to transition toward bone formation—a consideration that is critical when selecting cell types for specific regenerative applications.

### Chondrogenic spheroids from iMSCs show different characteristics in an *in vivo* setting

Following the observation that cNCC-iMSCs and SM-iMSCs exhibit higher chondrogenic potential, a qualitative *in vivo* study was conducted to examine the characteristics of iMSC-derived chondrogenic spheroids in a physiological setting. Briefly, chondrogenic spheroids were generated in a 3D culture system, subcutaneously transplanted into the dorsal region of immunocompromised mice, and collected after 8 weeks. To facilitate transplantation, a small incision was made on the backs of the mice, and the wound was sutured. Complete wound healing was observed within 1–2 weeks, and mice were in stable weight 5 weeks after procedure (Figure S17). Upon retrieval, the spheroids were cryopreserved, sectioned, and subjected to Alcian blue and von Kossa double staining to assess dystrophic calcification within the chondrogenic tissue. In the von Kossa test, silver ions (Ag^+^) replace calcium ions (Ca^2+^) within carbonate and phosphate salts, forming a pale-yellow silver precipitate. Exposure to strong light or ultraviolet (UV) radiation induces the photoreduction of silver salts, resulting in the deposition of metallic black silver (Figure 5C). Following double staining, cNCC-iMSC-derived chondrogenic samples remained negative for von Kossa staining, indicating the absence of mineralization (Figure 5D). In contrast, SM-iMSC-derived chondrogenic samples were positive for von Kossa staining, as evidenced by the presence of black precipitates. These findings further support the functional analysis in both 2D and 3D cultures, as well as the transcriptomic data from RNA-seq analysis, reinforcing the difference in chondrogenic and mineralization capacities between cNCC-iMSCs and SM-iMSCs.

### iMSCs exhibit distinct transcriptomic profiles reflective of their embryonic origin

**Figure 6:**
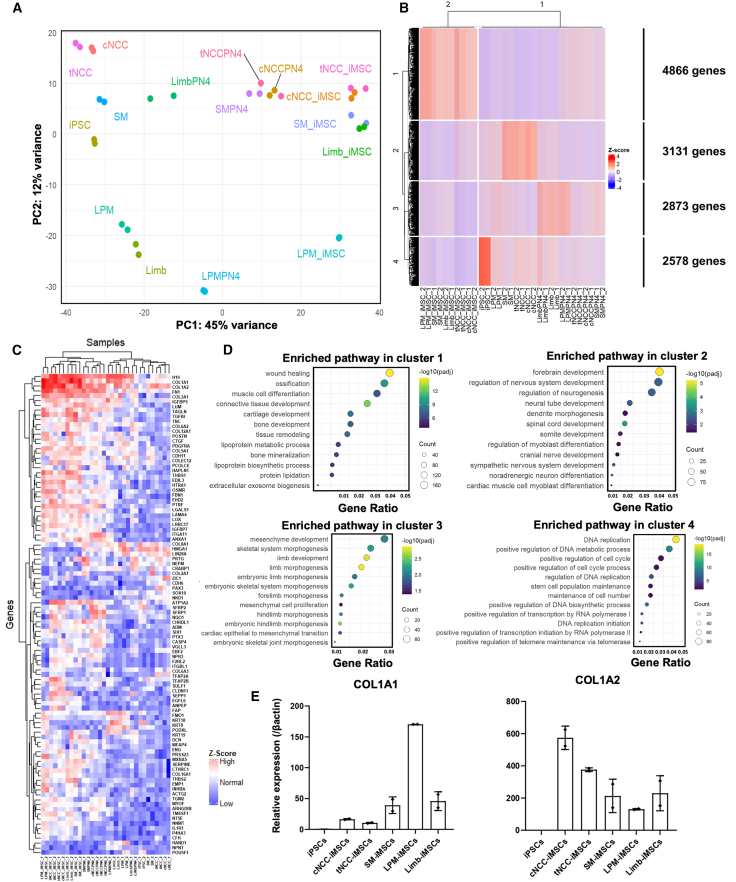
Transcriptomic profiling reveals distinct clusters among iMSCs derived from a single parental iPSC line (A) The PCA plot illustrates that the intermediate cell populations cluster according to their embryonic origins. The iMSCs form three distinct clusters: one cluster comprises cNCC-iMSCs and tNCC-iMSCs, another includes SM-iMSCs and Limb-iMSCs, and the third cluster consists of LPM-iMSCs. (B) A heatmap of global gene expression further supports these findings, displaying clustering patterns consistent with the PCA results. (C) Focusing on the top 100 most variably expressed genes during differentiation from iPSCs to expanded intermediates (PN4) to iMSCs, the heatmap aligns with the global expression patterns, highlighting significant changes in gene expression associated with the transition to iMSCs. (D) GO term analysis reveals enriched pathways within each cluster, which correspond to their known biological properties and functions. (E) qPCR results demonstrate that the expression levels of *COL1A1* and *COL1A2*, markers for mesenchymal stem cells, are significantly upregulated in the iMSC samples compared to the parental iPSCs. (Data are presented as mean ± SD, *n* = 2, independent experiments).

While differences in embryonic origin largely explain the distinct iMSC characteristics, we next examined the underlying gene expression profiles to identify additional regulatory pathways involved by conducting a comprehensive comparative analysis of the parental iPSCs, their intermediate progenitors in both initial and expanded states, and the corresponding iMSCs, comprising a total of 16 cell types (*n* = 2 each, 32 samples) (Figure S18A). Principal-component analysis (PCA) revealed that cNCCs and tNCCs formed a cluster, likely owing to their shared developmental origins (Figure 6A). Interestingly, these NCCs also clustered in close proximity to SMs, likely reflecting their shared embryonic origin and developmental trajectories.^69^^,^^70^ Furthermore, progenitors from the LPM and limb mesenchyme progenitors (Limb) cluster together, which is consistent with their shared developmental origins. Notably, when expanded under similar culture conditions, cNCC and tNCC (cNCCPN4 and tNCCPN4) groups were closer to SM (SMPN4), indicating that expanded SM exhibits a degree of plasticity, sharing significant transcriptional features with NCCs. However, SM-iMSCs clustered more closely with Limb-iMSCs than with NCC-iMSCs when subjected to the same MSC induction protocol. This suggests that the induction process using MSC medium may influence transcriptional changes in a mesodermal lineage-specific manner. In the context of iMSCs alone, three distinct clusters were identified: cranial and trunk NCCs, SM-iMSCs and Limb-iMSCs, and LPM-iMSCs. These clusters highlight the unique transcriptomic profile of iMSCs derived from different embryonic lineages and explain their distinct functional characteristics shown in the findings described above. Next, we generated a heatmap using the k-means clustering method applied to the normalized gene expression counts, which identified four primary clusters (Figure 6B). Cluster 1 consisted of 4,866 genes that were upregulated across all iMSCs and were slightly expressed in expanded SM, cranial, and trunk NCCs. This suggests transcriptional similarity between these expanded intermediates and their corresponding iMSCs. Cluster 2 consisted of 3,131 genes that were upregulated in SM and NCCs, consistent with the patterns observed in the earlier PCA analysis. Cluster 3 highlighted 2,873 genes that were upregulated in the expanded limb mesenchyme, limb progenitors, and expanded LPM, indicating shared gene expression profiles among these lineages. Finally, cluster 4 revealed 2,578 genes that were strongly expressed in iPSCs, distinguishing them from the other cell types analyzed. This heatmap further corroborated the clustering patterns observed in the earlier PCA plot, reinforcing the consistency between these two analytical approaches in identifying distinct clusters across different cell types. Furthermore, when the dataset was subject to a different method of data analysis, the number of clusters remained the same, with iPSCs, intermediates, expanded cells, and iMSCs, proving that the number of clusters is the same even when different analyses methods are used, indicating the sound biological rationale behind this clustering (Figure S18B). This was further supported by GO term analysis of genes highly expressed in iMSCs using this method, which revealed enrichment in MSC-related pathways such as ossification, chondrogenesis, protein lipidation, and exosome secretion (Figure S18C).

Based on these findings, we analyzed the 100 genes with the highest degree of differential expression across all 32 samples. The heatmap emphasizes genes that are specific to each cell type: *POU5F1* is highly expressed in iPSCs, whereas *HAND1*, *PAX3*, and *SOX10* are prominently expressed in the lateral mesoderm, paraxial mesoderm, and NCCs, respectively. This heatmap also reveals clustering between NCCs and SM-derived cells, as well as distinct groupings of cNCC-iMSCs and tNCC-iMSCs, and a cluster that includes both SM-iMSCs and Limb-iMSCs (Figure 6C). These patterns are consistent with the clustering observed in the heatmap of all genes and the earlier PCA plot, further validating the relationships between these cell types. To gain further insights into the upregulated genes within each cluster, we conducted GO term analysis (Figure 6D), which revealed enriched pathways corresponding to the known biological properties of each embryonic lineage. For instance, genes involved in bone and chondrogenic development and lipid metabolism were enriched in iMSCs, whereas the limb mesenchyme and cells expanded from this origin were enriched in pathways related to limb and skeletal system morphogenesis. We conducted qPCR to validate the RNA-seq results depicted by Figure 6C at the molecular level, which showed that the expression levels of *COL1A1* and *COL1A2*, key markers of MSCs, were significantly upregulated in all iMSC samples compared with the parental iPSCs (Figure 6E). This confirmed the successful differentiation of iPSCs into iMSCs and supported the transcriptomic data indicating a mesenchymal phenotype.

### Transcriptomic profiles show a high level of correlation between iMSCs and tMSCs, with greater osteogenic potential in iMSCs

**Figure 7:**
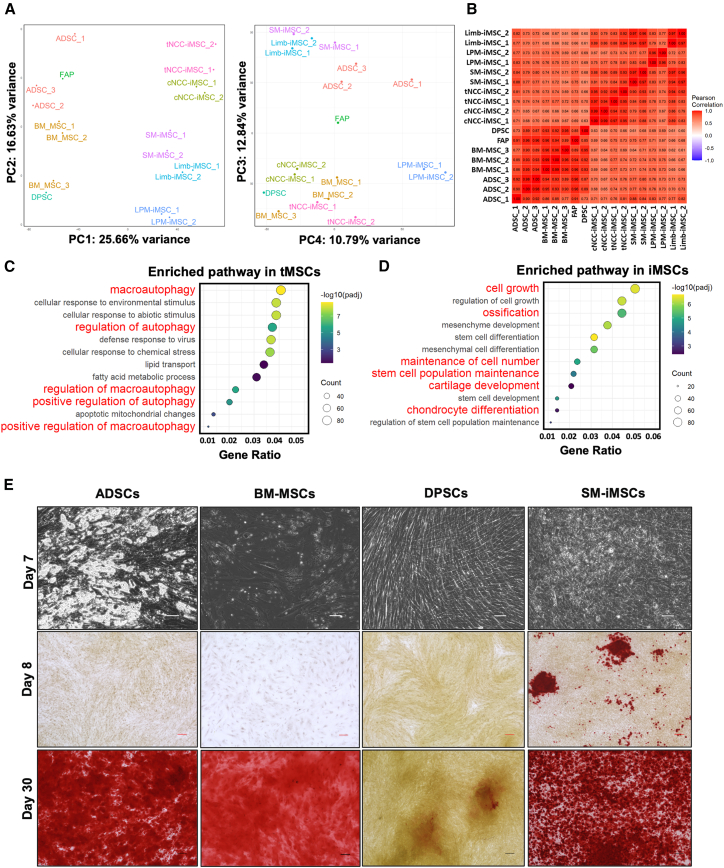
Transcriptomic profile comparison between iMSCs and tMSCs (A) PCA plot showing distinct clustering of iMSCs and tMSCs, and further analysis reveals close cluster of cNCC-iMSCs and DPSCs. (B) Correlation plot of global gene expression indicates a high correlation score between iMSCs and tMSCs, highlighting their transcriptomic similarities. (C) GO term analysis identifies enriched pathways in tMSCs, including macroautophagy and lipid metabolism. (D) Enriched pathways in iMSCs include those related to proliferation (e.g., cell growth and stem cell population maintenance) and differentiation (e.g., osteogenesis and chondrogenesis), suggesting a higher potential of iMSCs for osteoblast and chondrocyte differentiation. (E) Functional analysis reveals that SM-iMSCs show the earliest positive response to alizarin red staining (scale bars, 200 μm).

To examine transcriptomic differences between induced iMSCs and tMSCs, we performed a comparative global gene expression analysis of five distinct iMSC types with commonly studied tMSCs, including adipose tissue-derived MSCs (ADSCs), BM-MSCs, dental pulp-derived MSCs (DPSCs), and fibro-adipogenic progenitors (FAPs). PCA revealed a distinct clustering of iMSCs and tMSCs, suggesting variations in global gene expression patterns (Figure 7A). This finding aligns with a previous study by Lee et al.,^71^ which found differences in global gene expression between iMSCs and tMSCs. Further pairwise analysis of all other five combinations among PC1, PC2, PC3, and PC4 (main principal components) did not reveal consistent or biologically meaningful clustering patterns, with the exception of the PC3 and PC4 combination. This pairing revealed that NCC-derived iMSCs clustered moderately with BM-MSCs and DPSCs, suggesting some transcriptomic similarity among these populations. Given the established developmental link between DPSCs and cNCC-iMSCs—both originating from cNCC cells, which contribute to the formation of the single set of human teeth—GO enrichment analysis was performed on their highly expressed genes. This analysis revealed enrichment in odontogenesis-related pathways, including osteoblast/odontoblast differentiation and connective tissue development (Figure S19). These findings highlight the functional similarity and shared craniofacial differentiation potential of cNCC-iMSCs and DPSCs.

Despite the differences between iMSCs and tMSCs highlighted by PCA analysis, the correlation plot using Pearson clustering indicated a high degree of similarity (≥0.6) between the two cell types, implying that iMSCs share many molecular characteristics with tMSCs (Figure 7B). To dissect the functional implications of these transcriptomic similarities and differences, we conducted GO term enrichment analyses by putting all the iMSCs into one group and all tMSC samples into another. The result of these analyses highlighted that tMSCs exhibited enhanced pathways related to macroautophagy and positive regulation of autophagy (Figure 7C). This suggests limited expansion of tMSCs, as macrophagy and autophagy are related to cellular degradation, cell-cycle arrest, and senescence.^72^^,^^73^ In contrast, iMSCs showed enrichment in pathways associated with cell proliferation, growth, and stem cell population maintenance, as well as osteogenesis and chondrogenesis (Figure 7D). These findings suggest that iMSCs have a higher proliferative capacity and greater potential for differentiation into osteoblasts and chondrocytes than tMSCs.

To validate the functional implications of these transcriptomic differences, we assessed the osteogenic potential of both cell types. Functional assays demonstrated that SM-iMSCs initiated mineralization earlier than other groups, as evidenced by positive alizarin red staining at days 8 and 30; all tMSCs and SM-iMSCs showed positive staining at day 30, consistent with defined MSC characteristics (Figure 7E). Interestingly, similar to BM-MSCs, ADSCs displayed heterogeneity with evidence of adipogenic drift during osteogenic induction, possibly due to PPARG highly expressed subpopulation in ADSCs, indicated by lipid-droplet-containing cells (Figure 7E). These results underscore the enhanced osteogenic potential of iMSCs, particularly those derived from SMs, and highlight their potential applications in bone regeneration and repair.

## Discussion

This study investigated whether embryonic lineages influence the characteristics of iMSCs with the aim of contributing to the development of more effective and standardized MSC-based therapies. Detailed studies on iMSCs derived from five specific lineages—cranial and trunk neural crest, paraxial mesoderm (SM), LPM, and limb mesenchyme—indicated that embryonic origin influences the morphology, proliferation, differentiation capacities, and immunoregulatory functions of iMSCs. The distinct functional properties and molecular signatures of these iMSC types emphasize the importance of lineage-specific differentiation in tailoring MSCs for therapeutic applications. The use of human iPSC-differentiated intermediates enables the generation of multiple embryonic lineage cell population—such as neural crest, paraxial mesoderm, LPM, and limb mesenchyme—from a single, genetically defined source under controlled conditions.^25^^,^^26^^,^^37^^,^^38^ This approach overcomes the ethical and practical barriers associated with obtaining ideal positive control cells—mesenchyme and neural crest from human embryo tissues—and eliminates inter-donor variability together with developmental heterogeneity inherent to tMSCs.^74^ By applying well-established, stepwise differentiation protocols,^25^^,^^26^^,^^37^^,^^38^ these lineage-specific intermediates serve as reproducible internal references that exhibit consistent marker expression and allow systematic comparison of iMSC properties across embryonic origins.

At the first glance of cells’ size and granularity, our FSC/SSC profiles suggest a size/complexity-phenotype relationship whereby larger, more complex iMSCs exhibit stronger immunomodulatory activation (e.g., IFN-γ-induced PD-L1) but slower proliferation, whereas smaller cells proliferate faster with lower immune-response signals; future studies will prospectively sort subpopulations by FSC/SSC and test these associations formally (e.g., Spearman correlation). To be specific, slow-proliferated LPM-iMSCs may be suitable for therapeutic applications requiring immunosuppression; however, more functional validations are required, and although LPM-iMSCs proliferate slowly, this limitation can be mitigated by prolonging expansion at the LPM-intermediate stage, increasing initial seeding density, and leveraging LPM-iMSC-derived exosomes.

Our finding that SM-iMSCs exhibited the greatest osteogenic potential aligned with the role of SMs in the formation of the axial skeleton during embryonic development. The enhanced mineralization and calcification observed in SM-iMSCs suggest that these cells may be particularly well suited for bone-regeneration applications. Additionally, their high proliferation rate indicates robust potential for expansion, which is advantageous for generating sufficient cell quantities for clinical use. However, the tendency of SM-iMSCs toward hypertrophy and calcification during chondrogenic induction, as indicated by COL10A1 expression and positive von Kossa staining, could be a limitation in cartilage-repair applications where long-term stability is required. In contrast, cNCC-iMSCs, which show high chondrogenic potential and low COL10A1 expression, are promising candidates for cartilage repair. Beyond subcutaneous assays, future work will assess cartilage repair *in vivo* using intra-articular osteochondral-defect and osteoarthritis models (immunodeficient mice) with intra-articular delivery of cNCC-iMSCs or chondrogenic spheroids, standardized readouts of engraftment, hyaline matrix formation, hypertrophy control (COL10A1 expression), integration, and structural/functional outcomes.

An important consideration in the differentiation of iMSCs is the reproducibility of findings across different iPSC lines. To address this, we replicated our experiments using the 1383D6 and original 1231A3 cell lines. This additional validation demonstrated high efficiency in generating intermediates (cNCCs, SM, LPM, and Limb) and consistent morphological and osteogenic potential among the derived iMSCs. These results further supported the robustness of our protocol and highlighted the potential generalizability of our findings (Figures S7, S9, and S12).

In conclusion, this study demonstrated that the embryonic origin of iMSCs can influence their morphology, proliferation, differentiation capacities, and immunoregulatory properties (Table S1). By generating iMSCs through five distinct lineage-specific pathways, we found that SM-iMSCs exhibited superior osteogenic and chondrogenic potential, limb mesenchyme-derived iMSCs excelled in adipogenic differentiation, and cNCC-iMSCs displayed stable chondrogenic capabilities. These lineage-specific functional properties underscore the need for specialized culture conditions to optimize iMSC expansion and function. Further, these findings were replicated with iMSCs derived from two different iPSCs cell lines. Additionally, our findings revealed that iMSCs may have advantages over tMSCs, including enhanced proliferative and differentiation potential, making them promising candidates for regenerative applications. Future studies should focus on refining differentiation protocols and further exploring the therapeutic potential of each iMSC type *in vivo*.

### Limitations of this study

Despite providing additional insights into iMSCs’ characteristics and transcriptomic profile, this study has certain limitations in its experimental design and scope that should be acknowledged. First, the conditions for all iMSC types were standardized to ensure consistency across samples. The expansion medium used for intermediates contained only a TGF-β inhibitor along with EGF and FGF2, which we found generally optimal for the expansion of NCCs and SM-iMSCs. However, these conditions were unsuitable for the expansion of the LPM and limb mesenchyme cells. Specifically, LPM cells continued to express cardiac markers during expansion in media containing EGF and FGF (Figure S6D) and the absence of Wnt signaling limited the proliferation of limb mesenchyme-derived cells, as Wnt pathways play a key role in maintaining mesenchymal progenitors.^75^^,^^76^ Flow cytometry (FSC/SSC) subsequently revealed greater heterogeneity in LPM-iMSCs than other iMSCs, and sorting subpopulations by cell size and complexity may clarify underlying mechanisms and guide optimization of the expansion medium.

While this study focuses on IFN-γ-inducible *PD-L1/IDO1*, assessing iMSCs’ future immunoregulatory applications will benefit from systematically evaluating lineage-specific immunogenicity—including antigen presentation/co-stimulatory profiles, co-culture with T cells, and functional assays—to determine how these features relate to clinical applications. Additionally, including iMSCs generated directly from iPSCs (without lineage priming) would provide a necessary benchmark to isolate the specific contribution of lineage priming. Second, our study demonstrated the critical role of RA signaling in tNCC differentiation, as shown by the upregulation of trunk-specific markers (*HOXB5*, *HOXB6*, and *HOXB8*). Xue et al.^32^ showed downregulation; the gold standard for proving homogeneity—single-cell RNA-seq (scRNA-seq)—was not performed, and therefore, the possibility of minor contamination by other NCC subpopulations (i.e., HOXB2- or HOXB4-positive population) cannot be fully excluded. While scRNA-seq data are not yet available, cNCC-iMSCs, SM-iMSCs, LPM-iMSCs, and Limb-iMSCs are expected to exhibit similar transcriptional profiles in each population. This expectation is based on the use of well-established markers—CD271, DLL1, KDR^+^PDGFRA^+^, and KDR^+^PDGFRA^−^, respectively—to purify their progenitor intermediates (NCC, SM, LPM, and Limb progenitors), thereby ensuring lineage specificity. Future studies using scRNA-seq are essential to confirm the purity and transcriptional identity of each iMSC population. To reduce heterogeneity and improve iMSCs' quality, purification process ultilizes lineage-marker sorting (e.g., CD271/DLL1/KDR ± PDGFRA) combined with FSC/SSC gating and negative selection against residual pluripotency markers. Third, *in vivo* functional testing of iMSCs remains challenging due to unpredictable differentiation and the lack of pure tMSC controls, which are inherently heterogeneous,^2^^,^^3^ posing a difficulty in direct comparison of iMSCs with their *in vivo* counterpart. We, therefore, relied on ISCT minimum criteria of defining MSCs^18^^,^^19^ for comparisons across embryonic origins. In addition, while our study did not explore the hematopoietic support potential of iMSCs, which varies by origin and is not a universal MSC trait, this would be an interesting direction for future studies. Fourth, incorporating rapid *in vitro* functional assays—such as the scratch (wound-closure) test and the Boyden chamber/Transwell chemotaxis assay—to quantify iMSC motility would better inform future investigations of their therapeutic applications. Last, while *in vivo* transplantation is the gold standard for evaluating iMSCs’ functionalities,^77^ future studies may build upon these findings by exploring lineage-specific media formulations, incorporating single-cell transcriptomic profiling and integrating *in vivo* assays and metabolic analysis to further validate and enhance the developmental and therapeutic relevance of lineage-specific iMSCs.

## Resource availability

### Lead contact

Further information and requests for resources and reagents should be directed to and will be fulfilled by the lead contact Makoto Ikeya (ikeya-g@cira.kyoto-u.ac.jp).

### Materials availability

All materials are available from the corresponding author on reasonable request.

### Data and code availability

• RNA-seq data generated in this study have been deposited to GEO: GSE310465 and are publicly available as of the date of publication. Other data and images that support the findings of this study are available on request from the lead contact.
• This paper does not report original code.
• Any additional information required to reanalyze the data reported in this paper is available from the lead contact upon request.

## Acknowledgments

We sincerely thank Dr. Kanae Mitsunaga from the Core Laboratory, CiRA, 10.13039/501100005683Kyoto University, for her assistance with the FACS analysis. We also sincerely thank the former and current members of the Ikeya laboratory for their valuable support, especially Chengzhu Zhao, Daisuke Kamiya, Taiki Nakajima, Chiaki Mihara, Yoshiko Inada, Nakako Shimadzu, William Theoputra, and Nathalie Eileen Wiguna. This work was supported by the iPS Cell Research Fund; grants-in-aid for scientific research from the 10.13039/501100001691Japan Society for the Promotion of Science (10.13039/501100001691JSPS) (nos. 16H05447 and 23H03028); 10.13039/100009619Japan Agency for Medical Research and Development (10.13039/100009619AMED) under grant numbers JP22bm0104001, JP23bm1323001,and JP25ym0126808; KOSÉ Cosmetology Research Foundation; and Arktus Therapeutics to M.I. This work was also supported by a 10.13039/501100001700MEXT scholarship to N.L.

## Author contributions

Conceptualization, M.I.; funding acquisition, M.I.; investigation, N.L., S. Motoike, and M.I.; methodology, N.L., S. Motoike, D.Z., and M.I.; software: N.L., formal analysis: N.L., project administration, M.I.; resources, N.L., K.Y., Y.T., A.U., K.F., S. Maruyama, Y.J., J.T., and H.S.; data curation, N.L.; visualization, N.L.; N.L. performed all the experiments, produced all images and illustration including the graphical abstract, analyzed all the data, and performed all RNA-seq analysis; supervision, M.I.; writing – original draft, N.L.; writing – review and editing, N.L., D.Z., and M.I.

## Declaration of interests

This study was funded by iPS Cell Research Fund, JSPS, AMED, KOSÉ Cosmetology Research Foundation, MEXT, and Arktus Therapeutics. The funders were not involved in the study design, collection, analysis, interpretation of data, writing of this article, or the decision to submit the article for publication.

The authors declare the following competing interests: M.I. is a director of and receives compensation from Arktus Therapeutics. S.M. and J.T. serve as advisors to Arktus Therapeutics. M.I. is an inventor on patent applications related to the methods described in this manuscript: “Stepwise Method for Producing Various Cells from Pluripotent Stem Cells” (WO 2019/177118) and “Method for Producing Neural Crest Cells Specialized for Differentiation into Mesenchymal Lineage” (WO 2023/106122).

## Declaration of generative AI and AI-assisted technologies in the writing process

During the preparation of this work, the author (N.L.) used Grammarly and ChatGPT (subscribed) in order to check grammar and refine sentences. After using this tool/service, the author reviewed and edited the content as needed and takes full responsibility for the content of the publication. Icons in graphical abstract and figures have been created by BioRender (subscribed).

## STAR★Methods

### Key resources table

**Table:** 

REAGENT or RESOURCE	SOURCE	IDENTIFIER
**Antibodies**		

CD34 PE	Biolegend	343506
CD45 APC	Biolegend	368512
CD73 PE	BD Pharmigen	550257
CD90 APC	Biolegend	328113
CD105 APC	Invitrogen, eBiosciences^TM^	17-1057-42
CD271 Alexa fluor 647	BD Pharmigen	560326
DLL1 APC	R&D Systems	FAB1818A
CD309 PE	BD Pharmigen	560872
PDGFRα Alexa fluor 647	BD Pharmigen	562798
COLX	Invitrogen, eBiosciences	14-9771-80
SOX10	R&D Systems	AF 2864
p75	Santa Cruz Biotechnology	sc-271708
TWIST1	Santa Cruz Biotechnology	sc-81417

**Chemicals, peptides, and recombinant proteins**		

A83-01	Tocris	2939
C59	Cellagen Technology	C7641-2S
CHIR99201	Tocris	4423
DMH1	Tocris	4126
LDN193189	Stemgent	04-0074
PIK90	Millipore	528117-5MG
Retinoic acid	Sigma	R2625-50MG
SB-432542	Tocris	S1067
Activin A	R&D Systems	338-AC-050
BMP4	R&D Systems	314-BP-050
EGF	R&D Systems	236-EG
FGF2	R&D Systems	3718-FB
TGFβ-3	Peprotech Inc.	100-36E

**Deposited data**		

RNAseq data	This paper	GEO: GSE310465

**Experimental models: Cell lines**		

1383D6 iPSC line	Provided from Yamanaka laboratory, CiRA, Japan	–
1231A3 iPSC line	Provided from Yamanaka laboratory, CiRA, Japan	–

**Experimental models: Organisms/strains**		

NSG mice (male)	The Jackson Laboratory	–

**Oligonucleotides**		

Primers for RT-qPCR analysis, see Table S6	This paper	–

**Software and algorithms**		

R version 4.0 with compatible packages.	–	–

### Experimental model and study participant details

#### Human induced pluripotent stem cells (iPSCs), induced mesenchymal stem/stromal cells (iMSCs), and tissue-derived MSCs (tMSCs)

Two human iPSC lines 1383D6 (RIKEN BRC HPS1006; peripheral blood, male, 36 y; RRID: CVCL_UP39) and 1231A3 (RIKEN BRC HPS0381; peripheral blood, female, 29 y; RRID: CVCL_LJ39; expanded at the Center for iPS Cell Research and Application, Kyoto University) were cultured on iMatrix-511 (Nippi, Tokyo, Japan)-coated cell culture plates and dishes in StemFit AK03N (Ajinomoto, Tokyo, Japan). The ROCK inhibitor Y-27632 (Wako, Osaka, Japan) was added for 1 day after thawing or passaging cells. Human BM-MSCs used in Figures 2, 3, 4, 5, and 6, and DPSCs were purchased from Lonza (Basel, Switzerland). BM-MSCs used in Figure 7, ADSCs, and FAPs were provided by the Toguchida, Maruyama, and Uezumi laboratories, respectively. Both iMSCs and tMSCs were cultured on fibronectin (Millipore, Bedford, CA, USA)-coated six-well plates in PRIME-XV MSC Expansion XSFM medium (FUJIFILM Irvine Scientific, Tokyo, Japan). The cells were passaged every 3 days or when they reached 80% confluence. At each passage, iMSCs were assessed by Trypan Blue in triplicate; only cultures with ≥90% viability proceeded to downstream assays, and all lineages met this criterion. No mycoplasma contamination was confirmed using the MycoBlue Mycoplasma Detector (Cat# D101-02, Nippon Genetics Co.,Ltd).

#### Ethics statement

Studies involving human participants were reviewed and approved by the Ethics Committees of Kyoto University, Kyushu University, and Nagoya University. All the participants provided written informed consent to participate in this study. Ethical approval number is as below (G1052 for BM-MSC, CiRA19-12 for ADSC, and #55 for FAP).

#### Sample size and allocation

This study utilized primary human cells derived from adult donors. The sample size consisted of three ADSC lines, four BM-MSC lines, one FAP line, and one DPSC line. No randomization was performed for group allocation, as experimental groups were defined based on the specific tissue origin and cell type identity.

### Method details

#### Induction of five different intermediates and their iMSC counterparts

For induction of cNCCs, we used a previously well-established protocol by Kamiya et al., 2022. In short, human iPSCs were seeded onto iMatrix-511-coated plates or dishes at a density of 3.6 × 10^3^ cells/ cm^2^ in StemFit AK03N medium. After 4 days, the medium was changed to StemFit Basic03—which is equivalent to AK03N without FGF2 (both from Ajinomoto, Tokyo, Japan)—supplemented with 10 μM SB431542 (FUJIFILM Wako, Japan) and 1 μM CHIR99021 (Axon Medchem, Reston, VA, USA) and cultured for 10 or 11 days. Cells were counted using a Countess II FL device (Thermo Fisher Scientific). The medium was changed every 2 days from day 0 to day 6 and daily from day 6 onward. On day 10 or 11, cells expressing high levels of CD271 were sorted for further expansion. To induce tNCCs, the conditions were nearly identical to those used for cNCC induction, with the only change being the addition of 10 μM retinoic acid (Sigma Aldrich, Iowa, USA) to the medium starting on day 6. The medium was changed every 2 days from day 6 to 11. On day 11, the CD271^high^ cells were sorted for expansion. Both cNCCs and tNCCs were expandable in StemFit Basic03 medium supplemented with 10 μM SB431542, 20 ng/mL EGF (FUJIFILM Wako, Japan), and 20 ng/mL FGF2 (FUJIFILM Wako, Japan). The medium was replaced whenever the color turned orange. For passaging, the cells were dissociated using Accutase (Innovative Cell Technologies, San Diego, CA, USA) and replated onto fibronectin-coated six-well plates at a density of 1 × 10^5^ to 2 × 10^5^ cells/well. To prepare frozen stocks of NCCs, we collected 1 × 10^6^ NCCs from passage number 2 (PN2) or 4, suspended them in 1 mL of STEM-CELL BANKER GMP grade (Takara, Kusatsu, Japan).

To induce paraxial mesoderm (somites), we followed an established protocol by Nakajima et al., 2018. Briefly, iPSCs were seeded onto iMartix-511-coated 10-cm dishes at 5.6 × 10^4^ cells/dish and cultured in AK03N medium. After 3 days, we induced the differentiated of the iPSCs into presomitic mesoderm (PSM) by culturing them for 4 days in StemFit Basic03 medium supplemented with 10 μM SB431542 (Sigma, St. Louis, MO, USA), 10 μM CHIR99021 (Wako, Japan), 2 μM DMH1 (Tocris, Bristol, UK), and 20 ng/mL FGF2 (Wako, Japan). For somite differentiation, the collected PSM cells at day 4 were then replated onto iMatrix-511-coated 10-cm dishes at a density of 1.8 × 10^4^ cells/cm^2^ and cultured for an additional 4 days in StemFit Basic03 medium containing 10 μM SB431542 and 5 μM CHIR99021. Paraxial mesoderm (somite) cells were expanded using the same method as that used for NCCs, as described previously. These cells were preserved at passage number 2 (PN2) or 4 using the same techniques and reagents as for the NCCs.

To generate LPM and limb mesenchyme, we adapted previously published protocols by Loh et al., 2016 and Yamada et al., 2021 with several modifications. First, iPSCs were plated onto iMatrix-511-coated six-well plates at a density of 3.6 × 10^4^ cells/well and allowed to form colonies for 3 days. For differentiation into mid-primitive streak cells, we used StemFit Basic03 medium supplemented with 30 ng/mL Activin (R&D Systems, Minneapolis, USA), 40 ng/mL BMP4 (R&D Systems), 6 mM CHIR99021 (Axon Medchem, Reston, VA, USA), 20 ng/mL FGF2 (Wako, Japan), and 100 nM PIK90 (Millipore, Massachusetts, USA). The next day, the medium was replaced with medium supplemented with 1 μM A83-01 (Tocris, Bristol, UK), 30 ng/mL BMP4, 1 μM C59 (Cellagen Technology, San Diego, USA), and 6 μM Y-27632 (Wako, Japan) to promote LPM differentiation. Induction efficiency was assessed using FACS, and cells with high expression of KDR and PDGFRα were selected for further experiments. For expansion of intermediates, sorted cells were plated onto iMatrix-511-coated six-well plates at a density of 2× 10^5^ cells per well in StemFit Basic03 medium supplemented with 1 μM A83-01. Expanded cells were passaged every 3 days and cells were cryopreserved at passage number 2 or 4 in the same manner as intermediates from NCCs and SM.

For limb mesenchyme induction, sorted LPM cells were replated onto iMatrix-511-coated six-well plates at a density of 1.5 × 10^5^ cells per well. These cells were cultured in StemFit Basic03 medium supplemented with 1 μM A83-01 (Tocris, Bristol, UK), 0.5 μM LDN-193189 (Stemgent, Beltsville, MD), 3 μM CHIR99021, and 10 μM Y-27632. After 4 days, KDR^-^PDGFRA^+^ cells were sorted for further experiments. Limb mesenchyme progenitors were expanded as previously described.^27^ In short, sorted cells were plated onto iMatrix-511-coated six-well plates at a density of 2×10^5^ cells per well in a StemFit Basic03 medium supplemented with 1 μM A83-01, 3 μM CHIR99021, 20 μg/mL EGF, 20 μg/mL FGF2, and 10 μM Y-27632. Expanded cells were passaged every 3 days and cryopreserved at passage number 2 or 4 in the same manner as intermediates from NCCs, SM, and LPM. All reagents and proteins used to induce and maintain the intermediates are listed in Tables S3 and S4, respectively.

For iMSCs counterparts’ induction, the inductions were based similar to previous protocol^25^ as direct induction of iMSCs from iPSCs by medium switch can yield heterogeneous populations, we used lineage-specific differentiation with marker-based purification to constrain cell states and improve comparability across embryonic origins.^78^^,^^79^ Briefly, expanded intermediates were seeded onto fibronectin-coated or iMatrix-coated 6 well plate at a density of 1.5-2×10^5^ cells per well in Basic03 supplemented with appropriate expansion medium described previously. The medium was replaced the next day with PRIME-XV MSC Expansion XSFM medium (FUJIFILM Irvine Scientific, Tokyo, Japan). Passages were performed every 3-4 days using Accutase to dissociate cells. Human MSC markers (CD44, CD73, CD90, and CD105) together with negative markers (CD34, 45) were analyzed by FACS at passage number 5 (PN5) day 3.

#### Measurement of cell size and complexity

Cell size and internal complexity were assessed using FACS on a BD FACSAria II flow cytometer (BD Biosciences, Franklin Lakes, NJ, USA). FSC and SSC parameters were used to estimate cell size and granularity, respectively.^50^^,^^80^ FSC was used as a proxy for cell diameter or morphology, with larger cells exhibiting higher FSC values. SSC provides information on internal complexity, reflecting the presence of intracellular structures such as granules and organelles. A minimum of 10000 events were recorded per sample and data analysis was performed using FlowJo version 10. The samples were gated to exclude debris and doublets using the FSC-A/FSC-H parameter.

#### Flow cytometry

Flow cytometry analysis was performed using a BD Aria II instrument (BD Biosciences, Franklin Lakes, NJ, USA) following the manufacturer’s protocol. In summary, cells were dissociated with Accutase, incubated with specific antibodies in FACS buffer (Phosphate-Buffered Saline or PBS with 1% human serum albumin) for 60 min at 4°C, then washed again with FACS buffer before analysis. An isotype control was included in all experiments to minimize non-specific background signals. After incubation, the cells were washed with FACS buffer and resuspended in FACS buffer at a concentration of 1 × 10^6^ cells/mL Samples were then passed through a 35-μm filter (Falcon) and analyzed on the BD AriaII. Flow cytometry data were analyzed using FlowJo software (BD) ver 10.7.1. The antibodies used for FACS analysis and sorting are listed in Table S5.

#### Proliferation test

To compare the proliferation rates of iMSCs and BM-MSCs (control), all MSCs were plated on fibronectin-coated six-well plates at a density of 2 × 10^5^ cells per well in PRIME-XV MSC Expansion XSFM medium. Cells were collected and passaged every 3 days using COUNTESS II (Invitrogen, Thermo Fisher Scientific).

#### IFNγ treatment

All MSCs were plated at 1.5 × 10^5^ cells/well on a fibronectin-coated six-well plate.^25^ The cells were cultured 4 days to reach confluence. Following this, all iMSCs, along with BM-MSCs, were treated with 10 ng/mL IFNγ. Samples were collected exactly 24 h later and processed for qPCR analysis to assess the expression of specific immunoregulatory markers, *PD-L1* and *IDO1*.

#### Multi-lineage differentiation

Chondrogenic differentiation was achieved using micromass culture on gelatin-coated 24-well plates as previously described.^38^ Briefly, 1.5 × 10^5^ iMSCs were resuspended in 5 μL of chondrogenic medium composed of DMEM/F12 (Thermo Fisher Scientific, Waltham, MA, USA), 1% (v/v) ITS+ premix (Corning, Corning, NY, USA), 0.17 mM AA2P (Sigma-Aldrich, St. Louis, MO, USA), 0.35 mM Proline (Sigma-Aldrich), 0.1 mM Dexamethasone (Dex, Sigma-Aldrich), 0.15% (v/v) glucose (Sigma-Aldrich), 1 mM sodium pyruvate (Thermo Fisher Scientific), and 2 mM GlutaMAX (Thermo Fisher Scientific) supplemented with 10 ng/mL TGF-β3 (R&D Systems, Minneapolis, MN, USA) and BMP7 (50 ng/ml, R&D Systems). After 1 h, 1 mL of chondrogenic medium was added to each well, and the cells were cultured for 7 days. Chondrogenesis was evaluated by Alcian blue staining, fixed with 4% paraformaldehyde (PFA; FUJIFILM Wako), rinsed in phosphate-buffered saline (PBS), incubated with 1% Alcian Blue solution (Muto Pure Chemicals Co., Tokyo, Japan) for 1 h at room temperature (23-25°C), and washed five times with PBS before imaging. qPCR was performed to determine the expression of *COL10A1*, a specific marker of hypertrophy.^69^^,^^81^

For osteogenic differentiation, 5 × 10^4^ iMSCs were plated onto gelatin-coated wells, allowed to grow to full confluence, and cultured in osteogenic induction medium containing MEM-Alpha GlutaMAX (Gibco, 32571-036), 10% fetal bovine serum (Thermo Fisher Scientific), 0.5% penicillin/streptomycin, β-glycerophosphate disodium salt hydrate (Sigma-Aldrich, G9422), and 100 nM Dex. The medium was refreshed every 2–3 days.^25^ To detect mineralization, cells were fixed with PFA and stained with Alizarin Red S solution^76^ (Muto Pure Chemicals Co., Ltd., 17971) on days 7, 14, and 30.

For adipogenic differentiation, 5 × 10^4^ iMSCs were seeded in fibronectin-coated 12-well plates. After 6 days, adipogenic induction was initiated by replacing the MSC medium with adipogenic medium containing DMEM (08459-64, High Glucose; Nacalai Tesque, Japan), 10% fetal bovine serum (Nichirei, Japan), 0.5% penicillin/streptomycin (Gibco, USA), 10 μg/mL insulin (Wako Pure Chemicals Corp., Japan), 1 μM Dex (Wako Pure Chemicals Corp., Japan), 200 μM indomethacin (Wako Pure Chemicals Corp., Japan), and 500 μM IBMX (Wako Pure Chemicals Corp., Japan).^25^ Intracellular triglyceride droplets were detected using Oil Red O staining on day 28. Briefly, after washing twice with PBS and fixing with PFA, the cells were washed with distilled water, treated with 60% isopropanol for 5 min, and stained with Oil Red O (Nacalai Tesque, 25633-92) in 60% isopropanol. Excess stain was removed by washing several times with water.

#### Alizarin red quantification assay

For quantification of Alizarin Red staining,^76^ 800 μL of acetic acid (Nacalai Tesque Inc., Kyoto, Japan) was added to each well, and the plate was incubated at room temperature for at least 30 min with gentle shaking or until the monolayer was only loosely attached. The monolayer was then scraped from the plate using a cell scraper and transferred to a 1.5-mL microcentrifuge tube along with acetic acid. The sample was vortexed for 30 s, overlaid with 500 μL of mineral oil (Sigma–Aldrich) to avoid evaporation, and heated to exactly 85°C for 10 min, followed by cooling on ice for 5 min. To avoid sample loss, the tubes were not opened until fully cooled. The suspension was then centrifuged at 20,000 *g* for 15 min, and 500 μL of the supernatant was transferred to a new 1.5-mL microcentrifuge tube. Subsequently, 200 μL of 10% (v/v) ammonium hydroxide was added to neutralize the acid, and in some cases, pH was measured to ensure it was within the range of 4.1 to 4.5. The absorbance at 405 nm was measured using aliquots (150 μL) of the supernatant, in triplicate, in a 96-well plate with opaque walls and a transparent bottom (Thermo Fisher Scientific, USA).

#### Oil Red O quantification assay

Oil Red O was quantified by extracting the dye-stained triglyceride droplets inside the cells, following a previously established protocol.^35^^,^^36^ Briefly, after staining the samples with Oil Red O, 100% iso-propanol (Nacalai Tesque Inc., Japan) was added to each well (1 mL per well for 12-well plates and 2.5 mL per well for 6-well plates) to elute the stain, and the plates were gently shaken on an orbital shaker for 10 min at room temperature. From each well, 3 × 50 μL of the eluate was transferred to a clear polystyrene 96-well plate (100% iso-propanol used as negative control). Absorbance was measured at 510 nm using a multilabel reader (Envision, PerkinElmer, Waltham, MA, USA).

#### GAG assay

Micromasses of cells in 2D culture system were collected on day 7 and digested in 50 μg/mL papain solution (Sigma-Aldrich) at 65°C for at least 6 h or until the liquid become transparent. The obtained extracts were used to quantify the GAG content using Blyscan™—a sulfated GAG (sGAG) assay kit—following the manufacturer’s instructions.

#### qPCR

Total RNA was extracted using the RNeasy Micro Kit (Qiagen, Hilden, Germany) and reverse-transcribed into cDNA. Real-time quantitative PCR (RT-qPCR) was conducted using the THUNDERBIRD™ Next SYBR® qPCR mix (QPX-201; Toyobo Co., Ltd.), using the Step One Plus, QuantStudio™ 3 and QuantStudio™ 7 Flex Real-Time PCR Systems (Applied Biosystems, Waltham, MA, USA). The primer sequences are provided in Table S6. Data from at least two biological replicates were analyzed to determine relative fold changes using the 2^−ΔΔCT^ methods, and all results are presented as fold changes relative to the appropriate negative controls (iPSCs or fibroblasts) and visualized using GraphPad Prism 9.

#### 3D chondrogenic differentiation

For the 3D induction of chondrogenic spheroids from all iMSCs types and BM-MSCs, used as a control, we followed a previously described protocol.^66^ Briefly, 2 × 10^4^ cells/100 μL in chondrogenic medium were plated onto ultra–low attachment 96 U-well plates (Sumitomo Bakelite Co., Ltd., Tokyo, Japan) in hMSC Chondrogenic Basal Medium (Lonza, PT-3925) and hMSC Chondrogenic SingleQuots™ Kit Supplement (Lonza, PT-4121). Chondrogenic inducers were added at specific time points and important signals are indicated in Figure S11A. The cells were cultured for 21 days, and the medium was changed every 3–4 days. On day 21, spheroids were isolated and sectioned for Alcian blue staining and immunostaining.

#### Immunocytochemistry

Chondrogenic spheroids from iMSCs and BM-MSCs were collected on day 21 and preserved in 4% PFA. The spheroids were then embedded in OCT (Sakura Finetek, USA) and stored at −30°C for cryosectioning. Cryosections were cut at a thickness of 10 μm. The sections were subsequently incubated in Blocking One (Nacalai Tesque, Kyoto, Japan) at room temperature for 1 h to prevent non-specific binding. After blocking, the sections were incubated with an Anti-Collagen X antibody (Invitrogen, USA) at a 1:200 dilution in Blocking One for 1 h at room temperature. Next, the sections were incubated with the secondary antibody, donkey anti-goat IgG, Alexa Fluor 555 (Invitrogen, Carlsbad, CA, USA), at a 1:200 dilution at room temperature for 1 h. Nuclei were stained with DAPI (Thermo Fisher Scientific, USA) at a 1:200 dilution. Images were captured using a Keyence fluorescence microscope with consistent light exposure (2 s) for the red fluorescent signal (COL X) and 1/6s for the blue fluorescent signal (DAPI). The antibodies used for immunocytochemistry are listed in Table S7.

#### Transplantation experiment

Transplantation experiments were conducted according to a previously described protocol.^63^ Chondrogenic spheroids (day 21) from cNCC-iMSCs and SM-iMSCs were transplanted subcutaneously into the horizontal incisions on the backs of 8-week-old mice (male, NSG line, The Jackson Laboratory). The mice were anesthetized with 3% fluorane inhalant liquid (AbbVie, North Chicago, IL, USA). A small incision (3-4cm) was made at the back of the mice, spheroids were then transplanted by a pipet (optimal number 10-12). Wound was closed by nylon monofilament 4/0 suture, after that, mice were put in separate cages. After 8 weeks, the mice were sacrificed, and the spheroids were collected and sectioned (10–20 μm in thickness) for analysis. To visualize the calcium deposits in the cryosections, a modified von Kossa calcium staining kit (ScyTek Laboratories, UT, USA) was used according to the manufacturer’s instructions. The *in vivo* experiment was replicated 2 times. Ethical approval for the study was obtained from the Animal Care Committee of Kyoto University (16-73-13).

#### Double staining with von Kossa and Alcian blue

Double staining with von Kossa and Alcian blue was performed as previously described.^82^ At first, sections were stained with Alcian blue solution for 15 min, rinsed with distilled water, counterstained with Nuclear Fast Red for 3 min, and rinsed thoroughly to remove residual acid. For von Kossa staining, sections were rinsed in distilled water several times and then incubated with 1% silver nitrate solution in a clear glass Coplin jar under ultraviolet light for 30 min or in front of a 60–100-watt light bulb for at least 1 h. After silver nitrate treatment, the sections were rinsed in distilled water and unreacted silver was removed with 5% sodium thiosulfate for 5 min, followed by another rinse in distilled water.

#### RNAseq analysis

Total RNA was isolated using the RNeasy Micro Kit (Qiagen) and treated with the DNase I kit (Qiagen) to remove any residual genomic DNA. Then, 10 ng of total RNA was reverse-transcribed into single-stranded cDNA using the SuperScript VILO cDNA Synthesis Kit (Thermo Fisher Scientific). For NGS library construction targeting the Ion AmpliSeq Transcriptome, we utilized the Ion AmpliSeq Transcriptome Human Gene Expression Panel, Chef-Ready Kit (Thermo Fisher Scientific), and the Ion Chef (Thermo Fisher Scientific) instruments, following the manufacturer's protocol. Briefly, a reverse transcription master mix was prepared by combining 5X VILO Reaction Mix and 10X SuperScript III Enzyme Mix. The mix was then aliquoted into reaction plates, with 10 ng of total RNA added to each. The plates were loaded into a thermal cycler and subjected to incubation at 42°C for 30 min, followed by 85°C for 5 min, and then held at 10°C. During library preparation on the Ion Chef instruments, the following consumables and cartridges were loaded: Ion AmpliSeq Chef Solutions DL8 cartridge, Ion AmpliSeq Chef Reagents DL8 cartridge, Ion AmpliSeq Tip Cartridge L8, PCR Frame Seal, IonCode 96 Well PCR Plate, PCR Plate Frame, Empty Tip Cartridge L8, and Enrichment Cartridge. Notably, IonCode 96 Well PCR Plates used for reverse transcription reactions contained an IonCode barcode adapter. Additionally, the tube containing the Ion AmpliSeq Transcriptome Human Gene Expression Panel was inserted into an Ion AmpliSeq Chef Reagents DL8 cartridge. Multiplex PCR was performed in the thermal cycler within the Ion Chef, with 13 cycles of 16-minute annealing/extension reactions. Subsequent steps involved primer digestion with FUPA reagents, ligation of IonCode barcode adapters, library purification using magnetic beads, equalization of library concentrations, and mixing in a single tube. Following library preparation, the Ion Chef facilitated template preparation and ISP beads loading onto the Ion 540 Chip Kit. NGS sequencing was conducted using the Ion GeneStudio S5 Prime sequencer (Thermo Fisher Scientific). Count data were analyzed in Rstudio version 4.2. After filtering genes with low expression levels (mean ≤ 10), 13448 genes remained. The raw counts were then normalized and stabilized according to Anders and Huber (2010),^83^ who first introduced VST, which is recommended for medium-to-large datasets (e.g., more than 30 samples). Normalized counts were used for further analysis. Code for further experiments is provided in the Code Availability section.

### Quantification and statistical analysis

Statistical analyses were performed using GraphPad Prism software version 9 and RStudio. Data are collected from 2 or 3 independent experiments and presented as mean ± standard deviation (SD). A two-tailed t-test, followed by Welch’s correction or Bonferroni correction, was used to compare variables in the two groups of biological replicates. One-way analysis ANOVA with Tukey’s post hoc test was used to analyze differences among more than two groups. The results of all comparisons among the groups are presented in Table S2. Statistical significance was set at P <0.05. All results were collected from at least 2 independent experiments.

## Supplemental information

Document S1. Figures S1–S19 and Tables S1 and S3–S7

Table S2. All Tukey comparison tests (Excel file)
