# Supplementary material for: Embryonic lineage-specific iPSC-derived mesenchymal stem/stromal cells exhibit different morphologies and intrinsic functions
Source: iScience. 2025 Dec 18;29(1):114482. doi: 10.1016/j.isci.2025.114482 (PMC12803943; doi:10.1016/j.isci.2025.114482)
Supplement: Document S1. Figures S1–S19 and Tables S1 and S3–S7 [file mmc1.pdf]

iScience, Volume *29*

Supplemental information

Embryonic lineage-specific iPSC-derived mesenchymal stem/stromal cells exhibit different morphologies and intrinsic functions

Linh Nguyen, Souta Motoike, Denise Zujur, Keiko Yoshizawa, Yasuhiro Takashima, Akiyoshi Uezumi, Kazuhiro Furuhashi, Shoichi Maruyama, Yonghui Jin, Junya Toguchida, Hidetoshi Sakurai, and Makoto Ikeya

Figure S1

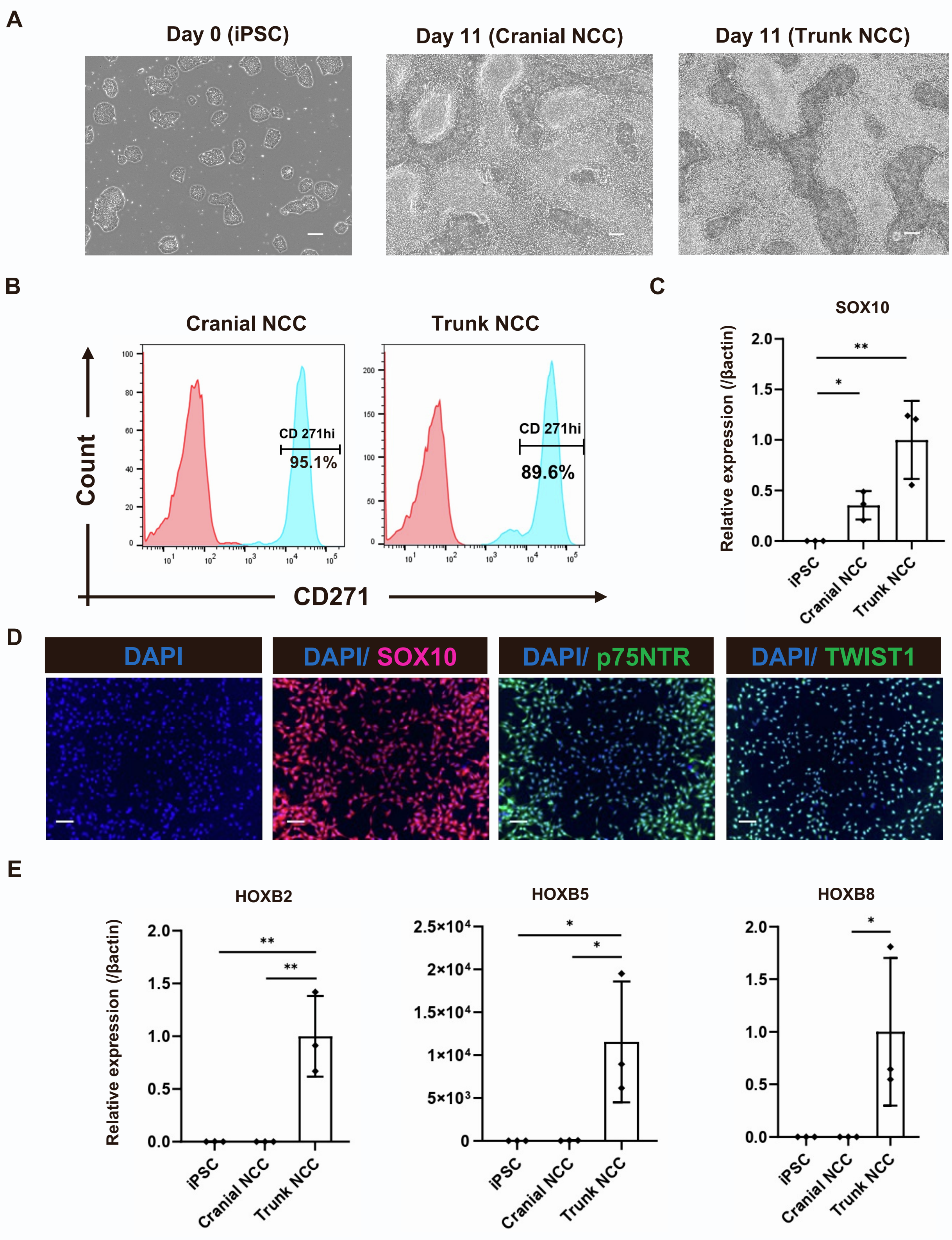

Figure S1. Generation and characterization of cranial and trunk neural crest cells, Related to Figure 1.
A) Phase-contrast images of iPSCs taken at Day 0 and during the induction process show cell growth reaching confluence by Day 11 for both cranial and trunk neural crest cell inductions (scale-bar: 200μm). B) Cranial and trunk neural crest cells were successfully generated with high efficiency by FACS analysis. C) Neural crest-specific marker *SOX10* was significantly upregulated in the CD271hi sorted population of both cranial and trunk neural crest cells. (Data are presented as mean ± SD, n = 3, independent experiments). D) Immunohistological staining revealed strong expression of additional NCC markers, including SOX10, p75NTR, and TWIST1 (scale-bar: 200μm). E) Trunk neural crest cells showed significant upregulation of trunk markers *HOXB2, HOXB5*, and *HOXB8* compared to cranial neural crest cells. (Data are presented as mean ± SD, n = 3, independent experiments, One-way ANOVA followed by Tukey's test)

Figure S2

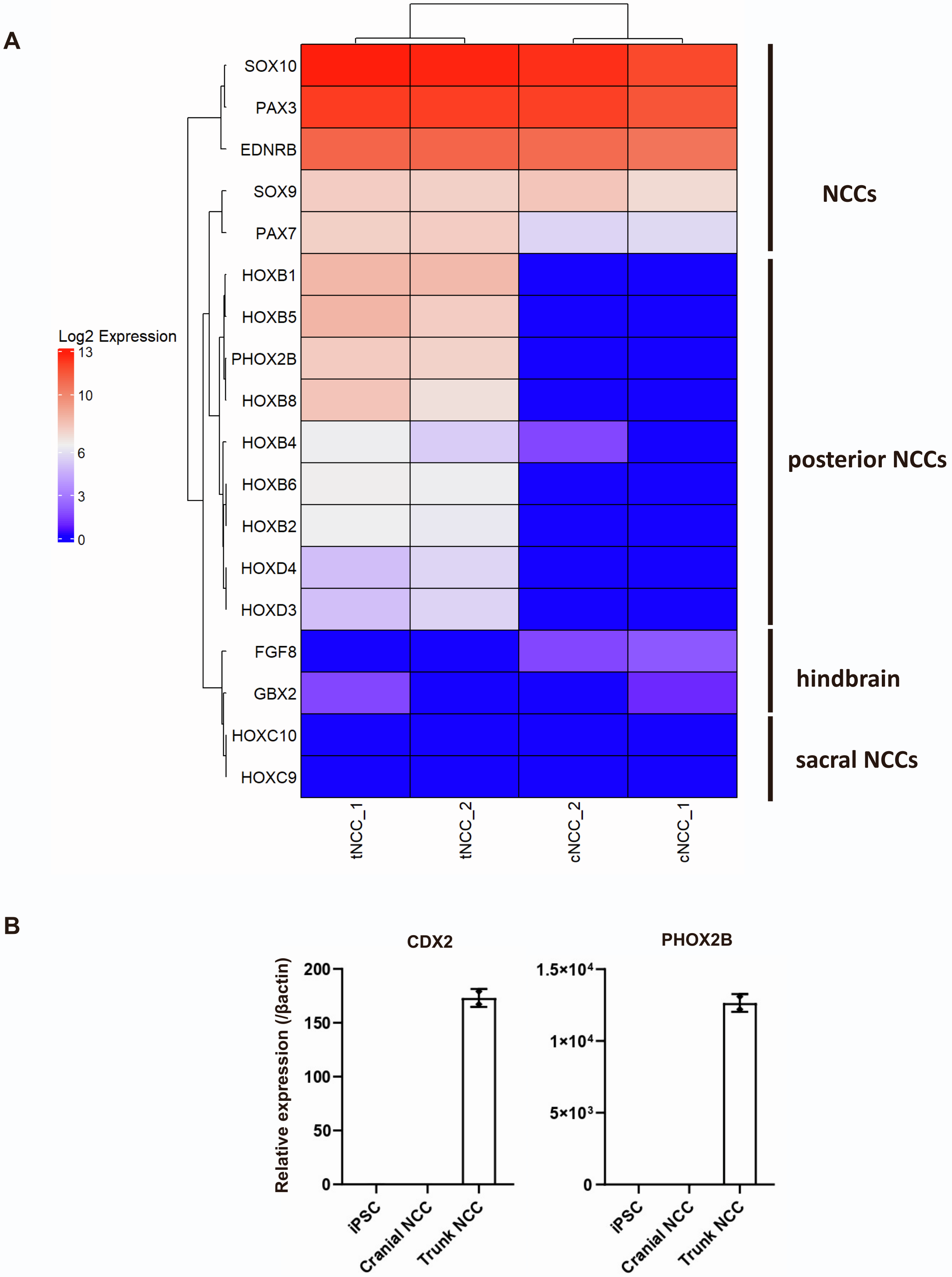

Figure S2. Transcriptomic profile of trunk neural crest cells, Related to Figure 1.
A) Heatmap analysis highlights the upregulation of trunk neural crest cell markers, while markers specific to cranial neural crest cells, hindbrain, and sacral NCCs were not expressed (n = 2, independent experiments). B) qPCR results further confirmed the upregulation of trunk neural crest cell markers such as *CDX2 and PHOX2B*. (Data are presented as mean ± SD, n = 2, independent experiments).

Figure S3

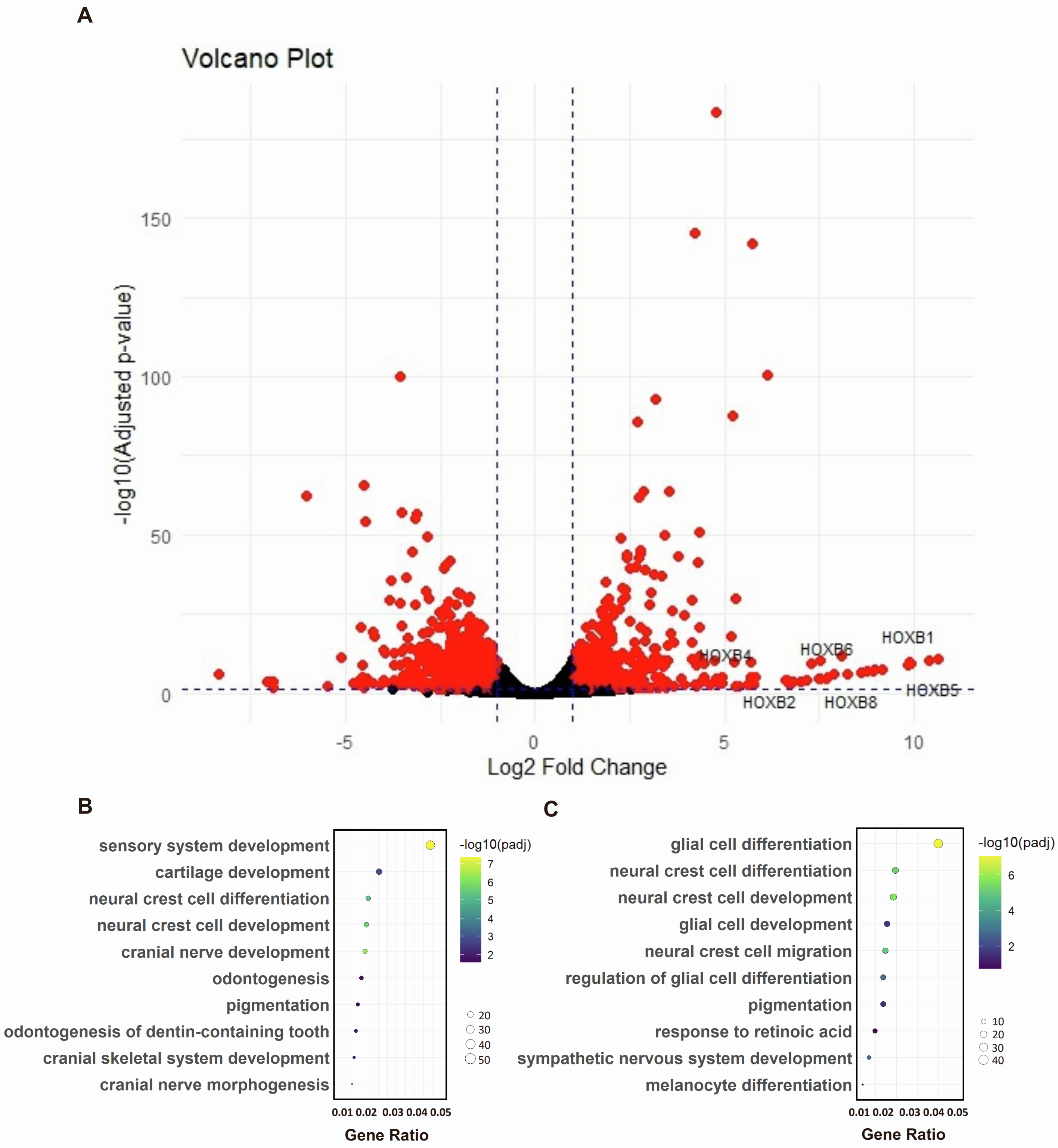

Figure S3. Transcriptomic profile of Cranial NCC and Trunk NCC samples, Related to Figure 1.
A) Volcano plot showed *HOXB* genes upregulated in trunk neural crest cells samples. B) Gene Ontology (GO) enrichment analysis of cranial neural crest cells reveals pathways related to cranial neural crest derivatives, including cranial nerve development, skeletal system development, and odontogenesis. C) GO enrichment analysis of trunk neural crest cells identifies enriched pathways associated with glial cell differentiation, melanocyte differentiation, and neural crest cell development, aligning with established result of cranial and trunk neural crest cells

Figure S4

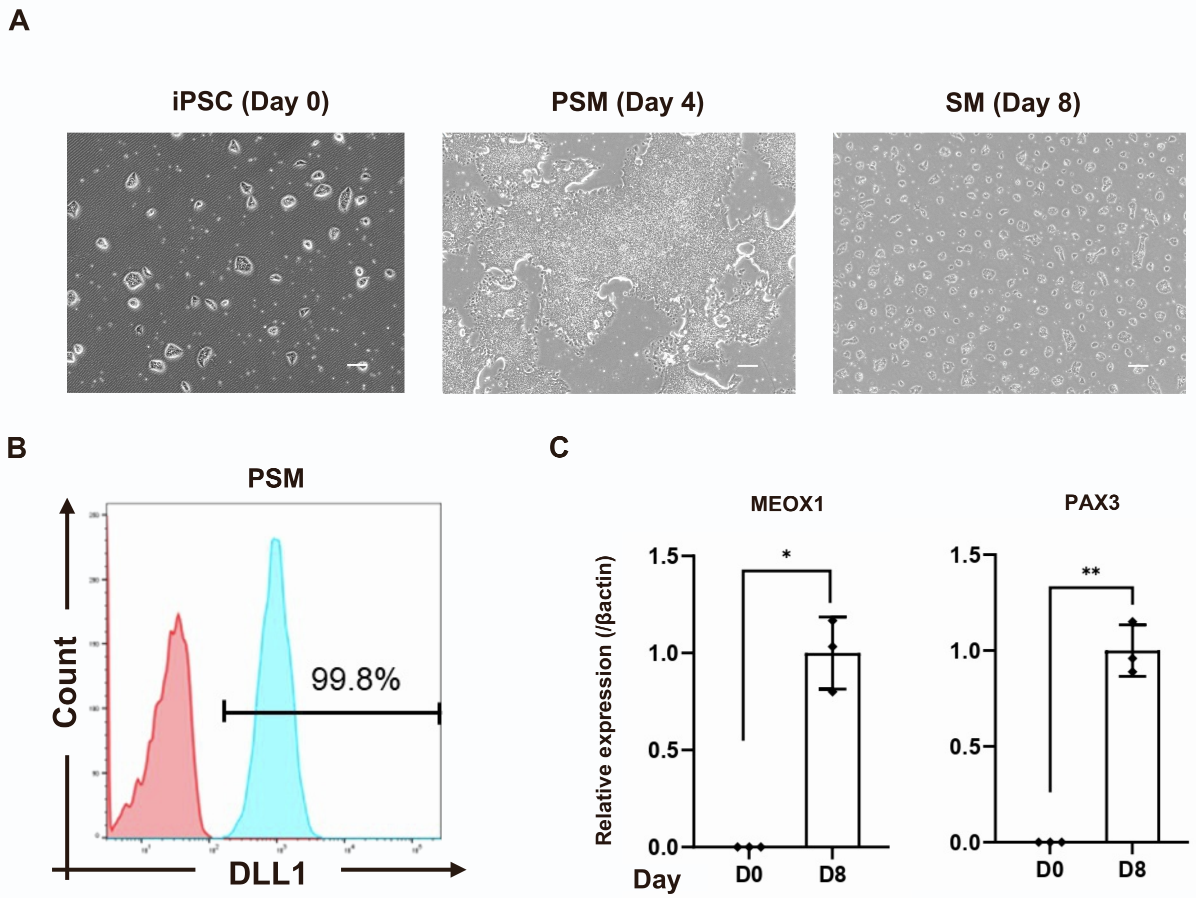

Figure S4. Successful generation of paraxial mesoderm (SM), Related to Figure 1.
A) Representative microscopy images depict the progression from iPSCs (characterized by compact colonies) to pre-somitic mesoderm (PSM) with larger, less-defined colonies, indicating differentiation at the edges, and finally to somites, which formed smaller, well-defined colonies post-passaging. B) Flow cytometry analysis of the generated PSM population shows over 90% expression of the key marker DLL1, confirming successful differentiation. C) qPCR analysis demonstrates significant upregulation of somite-specific markers *MEOX1* and *PAX3* (Data are presented as mean ± SD, n = 3, independent experiments, two-tailed t-test followed by Welch's correction).

Figure S5

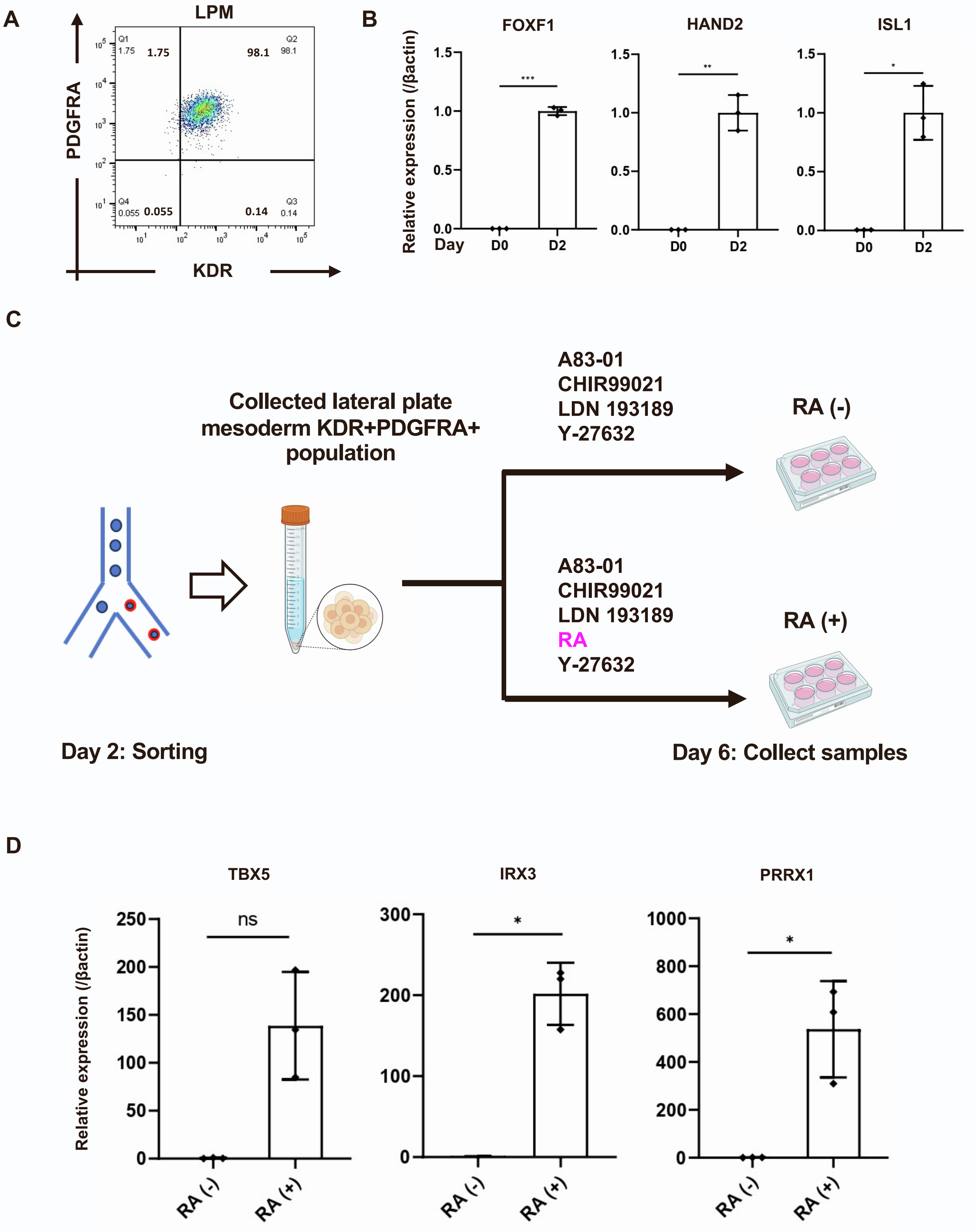

Figure S5. Generation and characterization of lateral plate mesoderm and limb mesenchyme from iPSCs, Related to Figure 1.
A) FACS analysis on Day 2 confirmed successful induction of lateral plate mesoderm, with over 90% of cells expressing KDR+PDGFRA+ markers. B) qPCR showed that other specific lateral plate mesoderm markers significantly upregulated. (Data are presented as mean ± SD, n = 3, independent experiments, Two-tailed t-test followed by Welch's correction). C) Lateral plate mesoderm cells (KDR+PDGFRA+) sorted at Day 2 were cultured in limb induction medium with or without retinoic acid for 4 days. D) qPCR data showed a significant upregulation in the expression of limb-specific markers (*TBX5*, *IRX3*, and *PPRX1*) in retinoic acid-treated cells compared to cells cultured without retinoic acid. (Data are presented as mean ± SD, n = 3, independent experiments, Two-tailed t-test followed by Welch's correction)

Figure S6

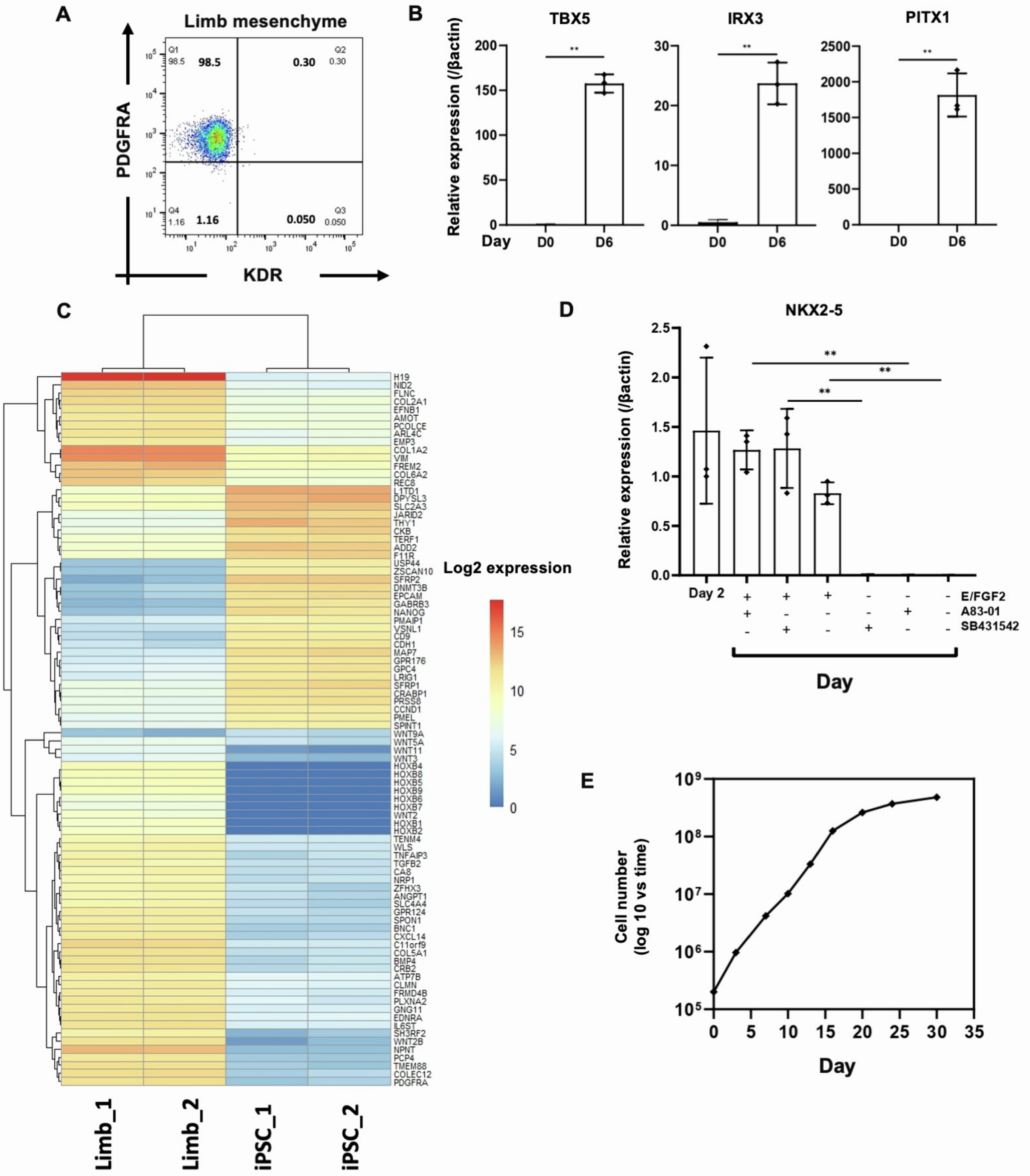

Figure S6. Successful generation of limb mesenchyme progenitors and expansion of lateral plate mesoderm, Related to Figure 1.
A) FACS analysis result demonstrated efficient induction of limb mesenchyme, as more than 90% of cells expressed limb-specific markers KDR-PDGFRA+. B) By Day 6, qPCR revealed significant upregulation of limb mesenchyme markers *TBX5, IRX3*, and *PITX1* in sorted cells (n = 3, independent experiments). C) A heatmap of RNA sequencing data showed increased expression of HOXB and Wnt pathway markers in limb mesenchyme samples (n = 2, independent experiments). D) *NKX2-5*, a cardiac-specific marker, was upregulated in lateral plate mesoderm samples expanded with EGF and FGF2 (n = 3, independent experiments, One-way Anova followed by Tukey's post hoc test). E) Lateral plate mesoderm cells showed sustained expansion in medium containing only A83-01.

Figure S7

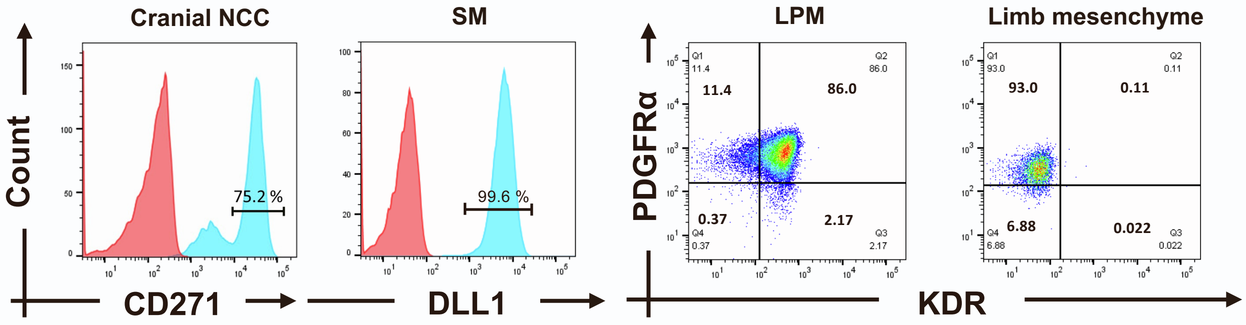

Figure S7. Generation of cranial neural crest cells (cNCCs), somite (SM), lateral plate mesoderm (LPM), and limb mesenchyme cells from the 1383D6 iPSC line, Related to Figure 1.

Specific markers were checked by flow cytometry analysis. The red histogram represents isotype controls, and the blue histogram shows samples stained with antibodies.

Figure S8

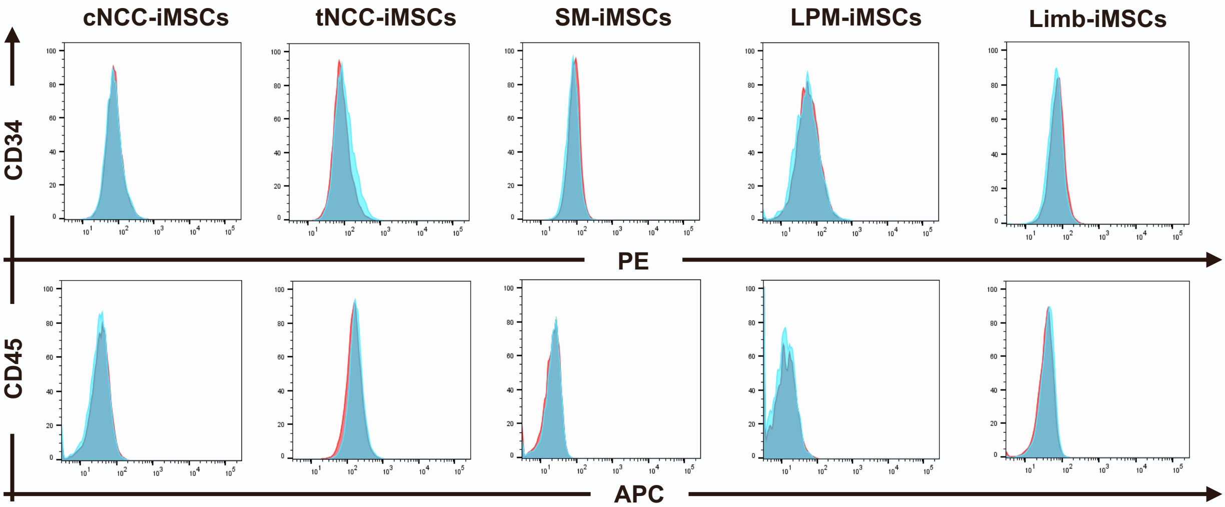

Figure S8. Negative marker analysis for iMSCs, Related to Figure 1.
FACS analysis showed negative markers CD34 and CD45 were not expressed in any type of iMSCs. The red histogram represents isotype controls, and the blue histogram shows samples stained with antibodies. (Representative data, n = 3, independent experiments).

Figure S9

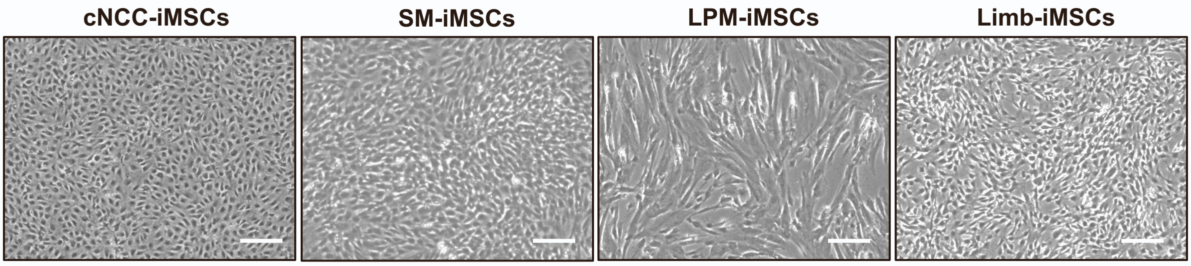

Figure S9. iMSCs derived from the 1383D6 iPSC cell line display variations in morphology, Related to Figure 2. Morphological diversity observed in iMSCs, with LPM-iMSCs exhibiting elongated, spindle-shaped cells, while other iMSC types show a smaller shape (scale-bar: 200μm).

Figure S10

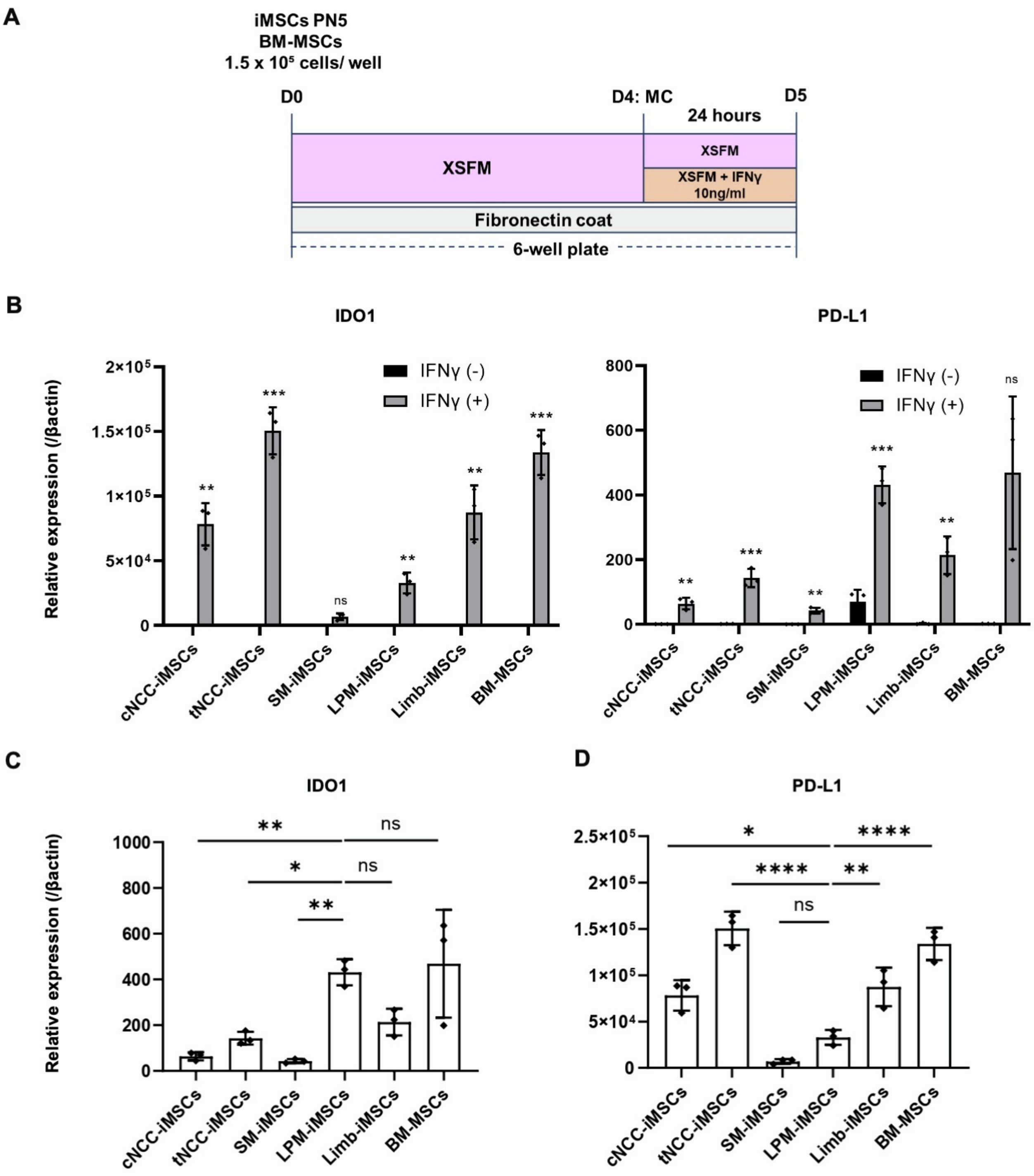

Figure S10. Immunoregulatory potential of all iMSCs, Related to Figure 2.
A) Schema of IFNγ treatment of all iMSCs with BM-MSCs as control, medium changed at Day 4 after plating the cells. B) Immunoregulatory markers *PD-L1* and *IDO1* were significant upregulation after 24 hours of IFN-γ treatment (Data are presented as mean ± SD, n = 3, independent experiments; statistical analysis performed using one-way ANOVA with post hoc Tukey's test). C) Expression levels of immunoregulatory markers varied among the iMSC types, with SM-iMSCs displaying the lowest levels. (Data are presented as mean ± SD, n = 3, independent experiments; statistical analysis performed using one-way ANOVA with post hoc Tukey's test, full results of all pairwise comparisons are provided in Supplementary table S2, p value notification: * : p<0.05; ** : p<0.01; *** : p<0.001). D) Notably, LPM-iMSCs expressed *PD-L1* without IFN-γ treatment (Data are presented as mean ± SD, n = 3, independent experiments; statistical analysis performed using one-way ANOVA with post hoc Tukey's test, full results of all pairwise comparisons are provided in Supplementary table S1, p value notification: * : p<0.05; ** : p<0.01; *** : p<0.001)

Figure S11

A

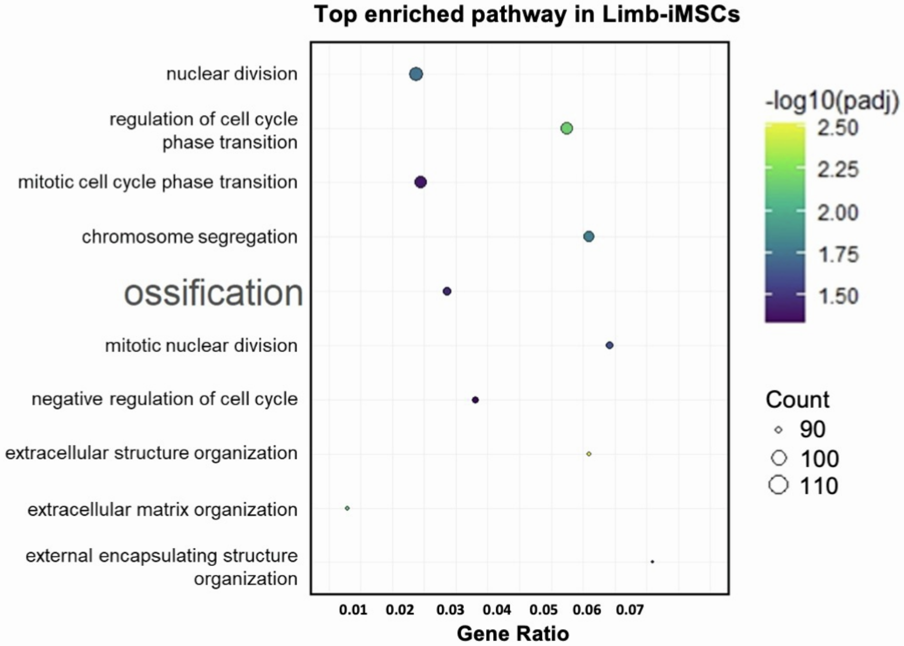

B

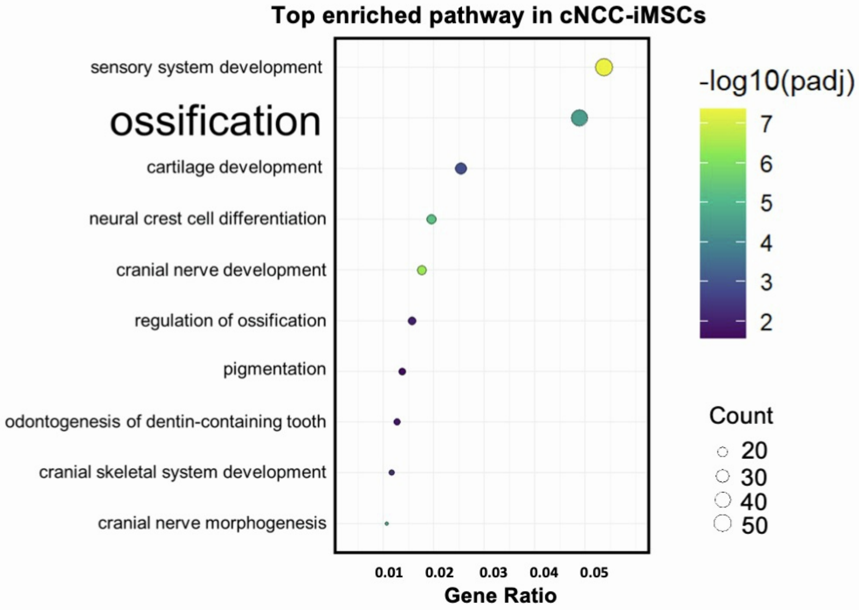

C

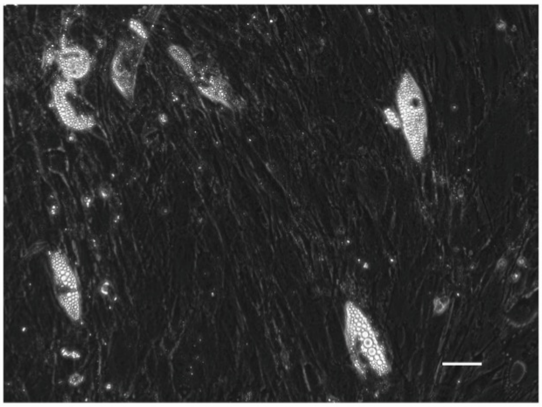

Figure S11. Transcriptomic and functional analysis of iMSCs highlights enriched osteogenesis-related pathways, Related to Figure 3.
A) GO terms analysis shows that Limb-iMSCs are enriched in osteogenesis-related pathways compared to LPM-iMSCs. B) cNCC-iMSCs also enriched in the ossification pathway compared to tNCC-iMSCs, aligning with the functional analysis. C) Osteogenic induction of BM-MSCs reveals the presence of cells containing triglyceride droplets, indicating heterogeneity in osteogenic induction when using BM-MSCs (scale-bar 50μm).

Figure S12

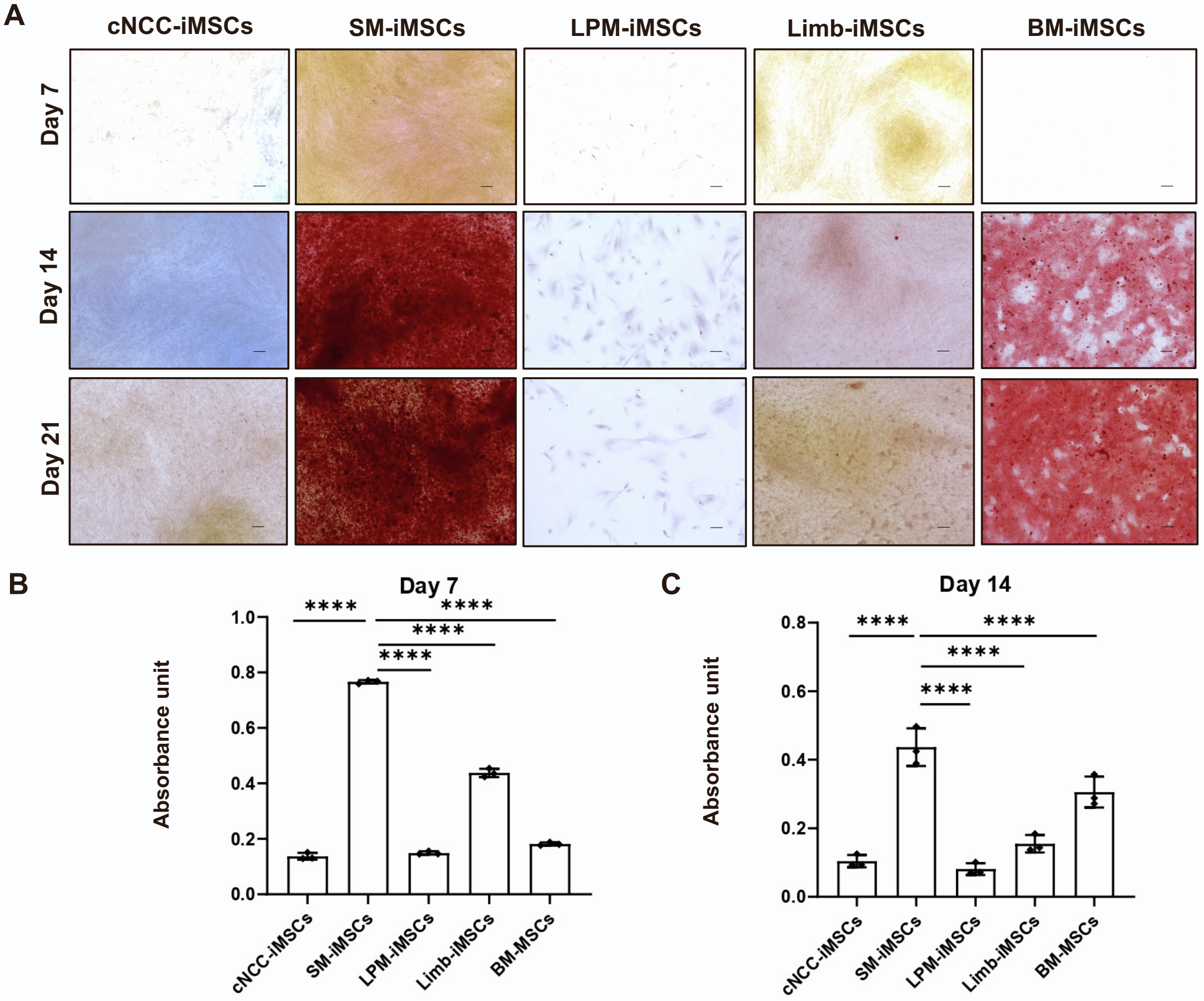

Figure S12. Differential osteogenic potential of iMSCs from 1383D6 cell line, with SM-iMSCs demonstrating the highest osteogenic differentiation, followed by Limb-iMSCs and cNCC-iMSCs, mirroring the differentiation patterns observed in iMSCs derived from the 1231A3 cell line, Related to Figure 3.

A) Quantification of mineralization by Alizarin Red staining at Day 7, showing the highest ossification in SM-iMSCs (Data are presented as mean ± SD, n = 3, independent experiments). B) Quantitative analysis of Alizarin Red staining indicates that SM-iMSCs have significantly higher mineralization compared to other iMSC types and bone marrow-derived MSCs (BM-MSCs), sample diluted by the 1:3 ratio when required at Day 7 and C) Day 14. (Data are presented as mean ± SD, n = 3, independent experiments, One-way ANOVA followed by Tukey's test; full results of all pairwise comparisons are provided in Supplementary Table S2, p value notification: * : p<0.05; ** : p<0.01; *** : p<0.001)

Figure S13

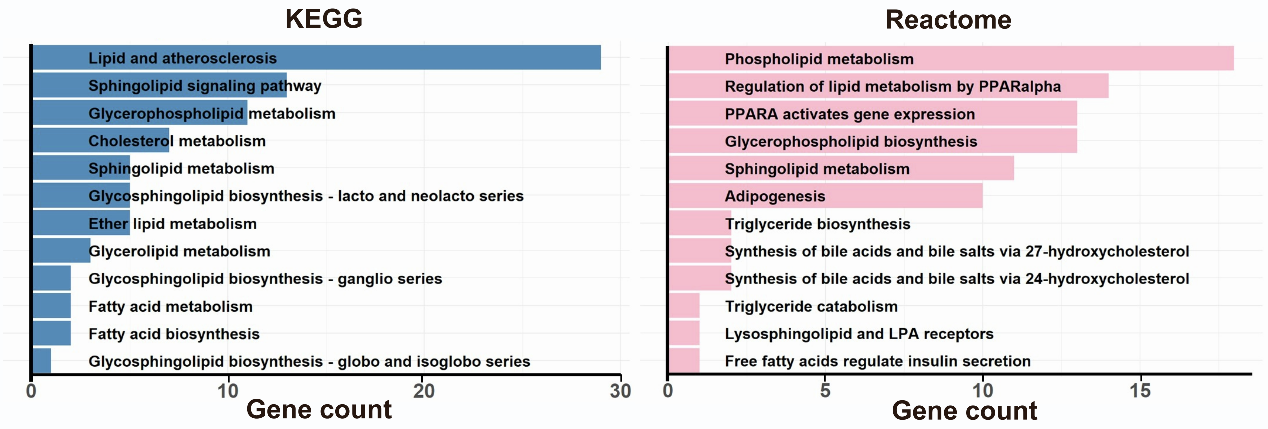

Figure S13. Upregulated genes in Limb-iMSCs are enriched in adipogenesis-related pathways compared to common databases such as KEGG and Reactome, aligning with functional and qPCR analyses, Related to Figure 4.

Figure S14

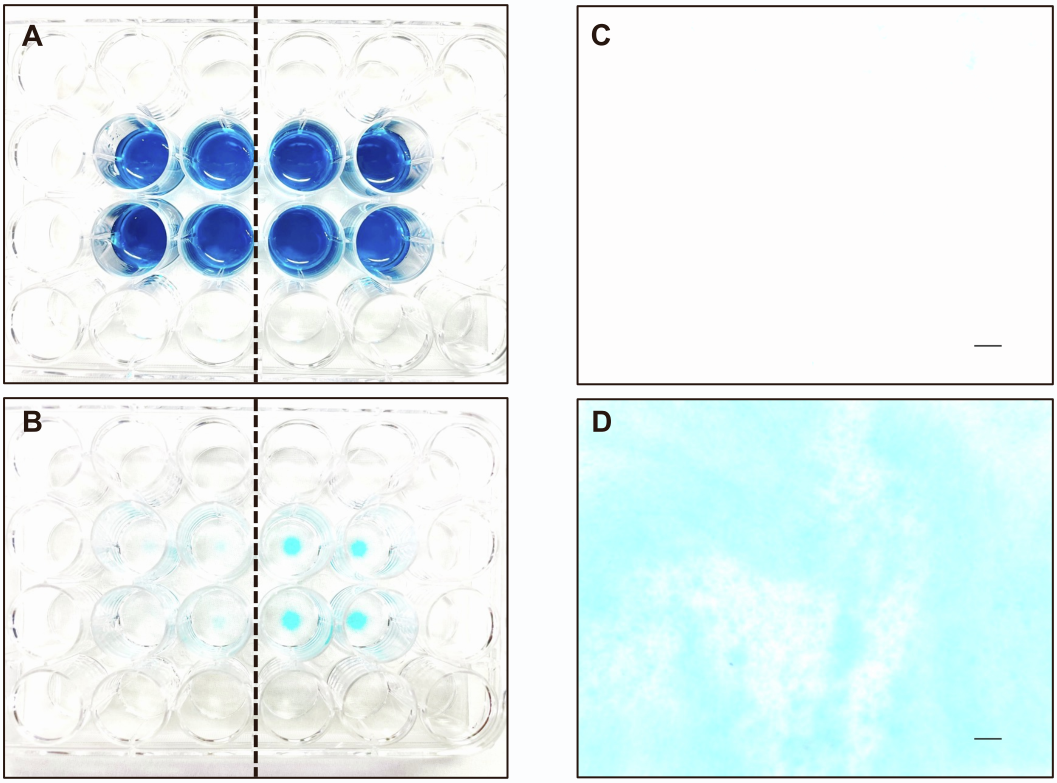

Figure S14. Negative control for Alcian blue staining in 2D chondrogenic culture, Related to Figure 4.
A, B) Negative control samples (cultures without key BMP and TGFβ signaling) show minimal or no Alcian blue staining (left), whereas positive control samples (right) exhibit strong Alcian blue staining, indicating successful chondrogenic differentiation. C, D) Microscopic images of negative (upper panel) and positive (lower panel) samples (scale-bar: 200μm).

Figure S15

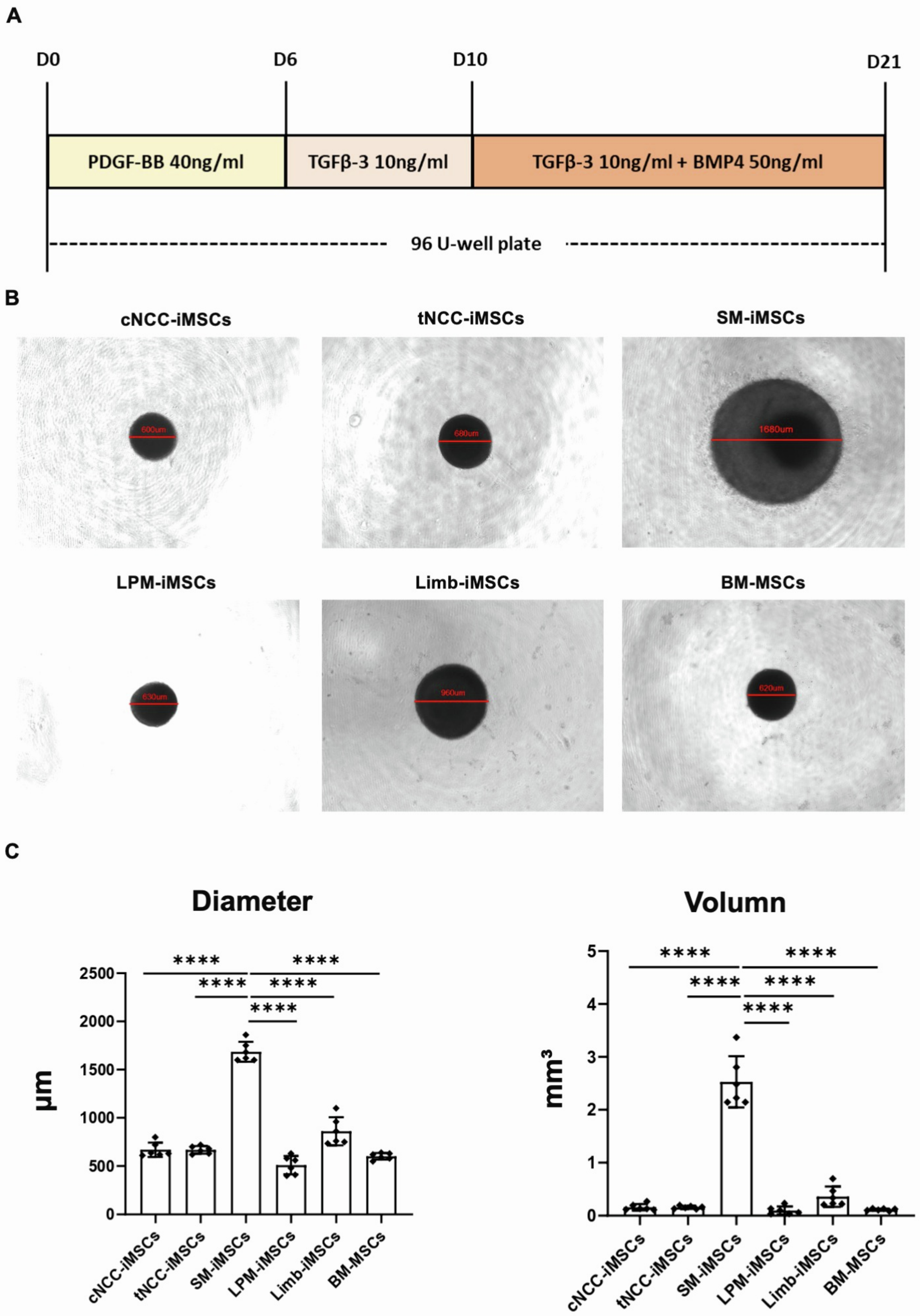

Figure S15. 3D chondrogenic differentiation of iMSCs, Related to Figure 5.
A) Schematic representation of the 3D chondrogenic differentiation process based on a previously established protocol. B) Phase-contrast microscopy images show variation in spheroid sizes among different iMSC types. C) Quantitative analysis using ImageJ reveals that chondrogenic spheroids derived from SM-iMSCs exhibit the largest size. (Data are presented as mean ± SD; n = 6 from 3 independent experiments; statistical analysis performed using one-way ANOVA, full result is provided in Supplemental table S2).

Figure S16

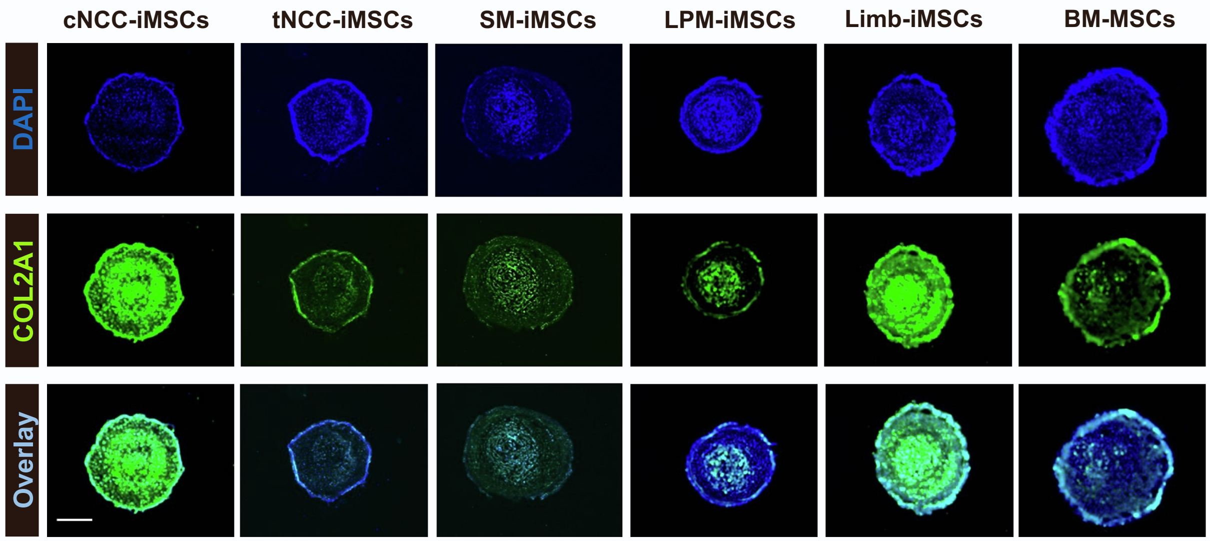

Figure S16. 3D culture of chondrogenic spheroids from iMSCs, Related to Figure 5.
Immunohistological analysis revealed COL2A1 expression in all chondrogenic spheroids (scale-bar: 200μm).

Figure S17

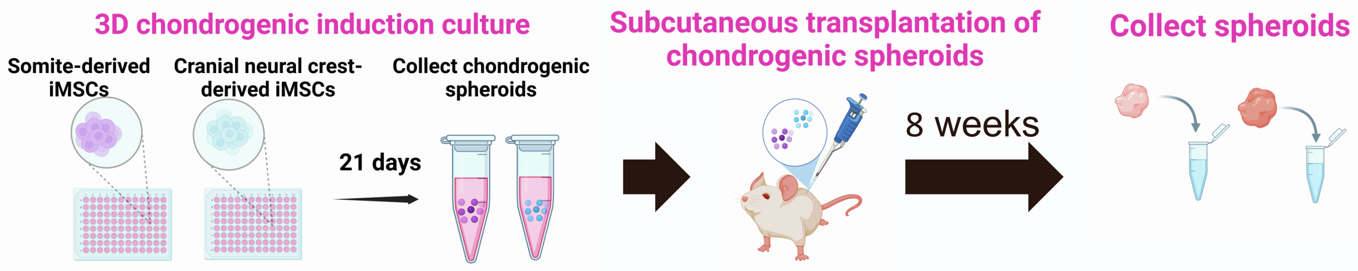

Figure 17. Chondrogenic spheroids from iMSCs showed different characteristics in *in vivo* setting, Related to Figure 5.
Schema of in vivo experiment.

Figure S18

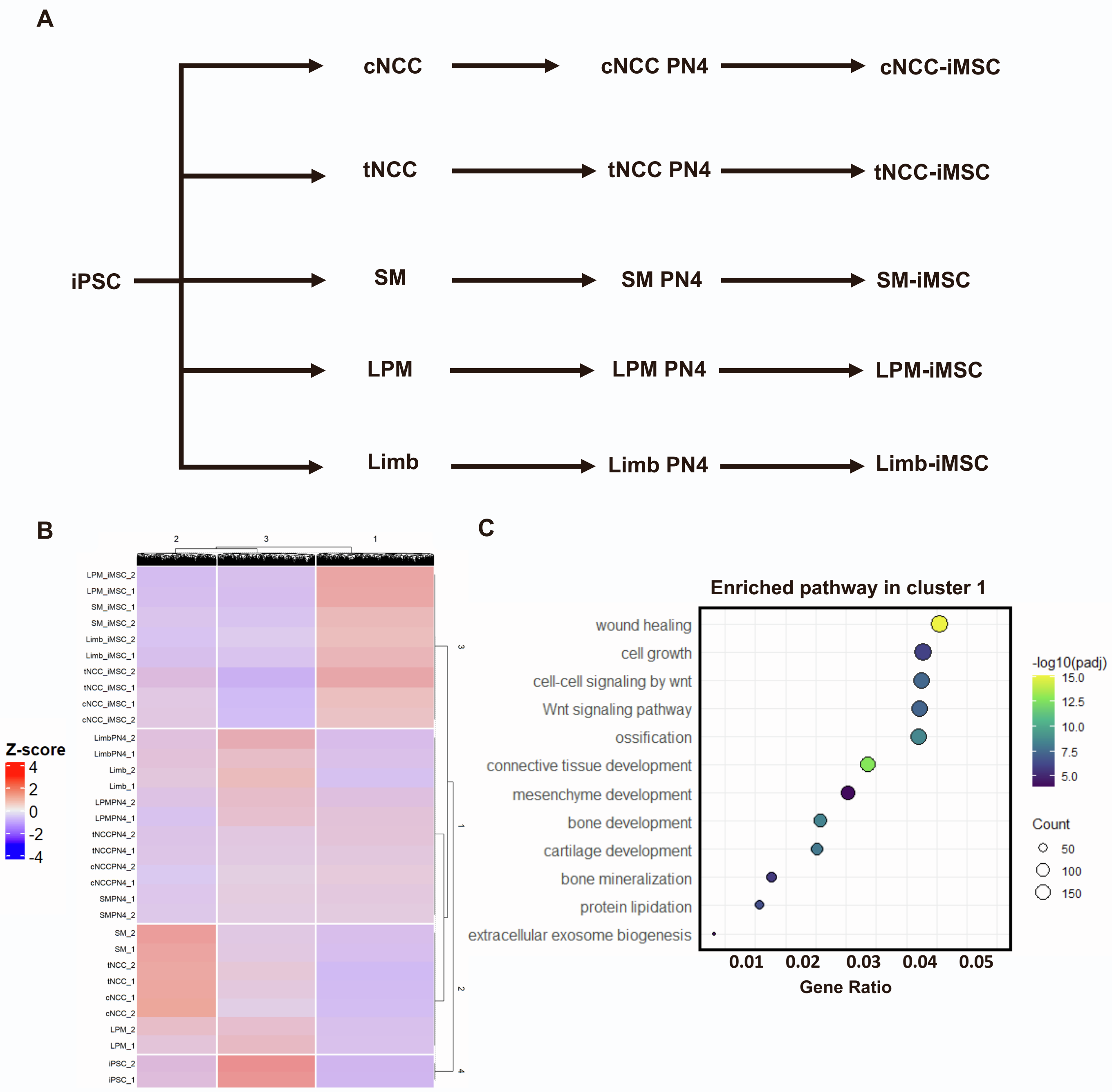

Figure S18. Transcriptional profiles of samples from iPSCs to iMSCs, Related to Figure 6.
A) Simplify schema that depicts 32 selected samples for RNA seq analysis from iPSCs to iMSCs (n=2, independent experiments). B) Clustering analysis using an alternative method (different defined number of samples' clusters) produced consistent results, grouping all iMSCs together, with iPSCs forming a separate cluster, intermediates in another, and their expanded counterparts in a distinct cluster. C) Pathway enrichment analysis revealed that iMSCs are enriched in pathways related to mesenchymal stem cell characteristics and the Wnt signaling pathway.

Figure S19

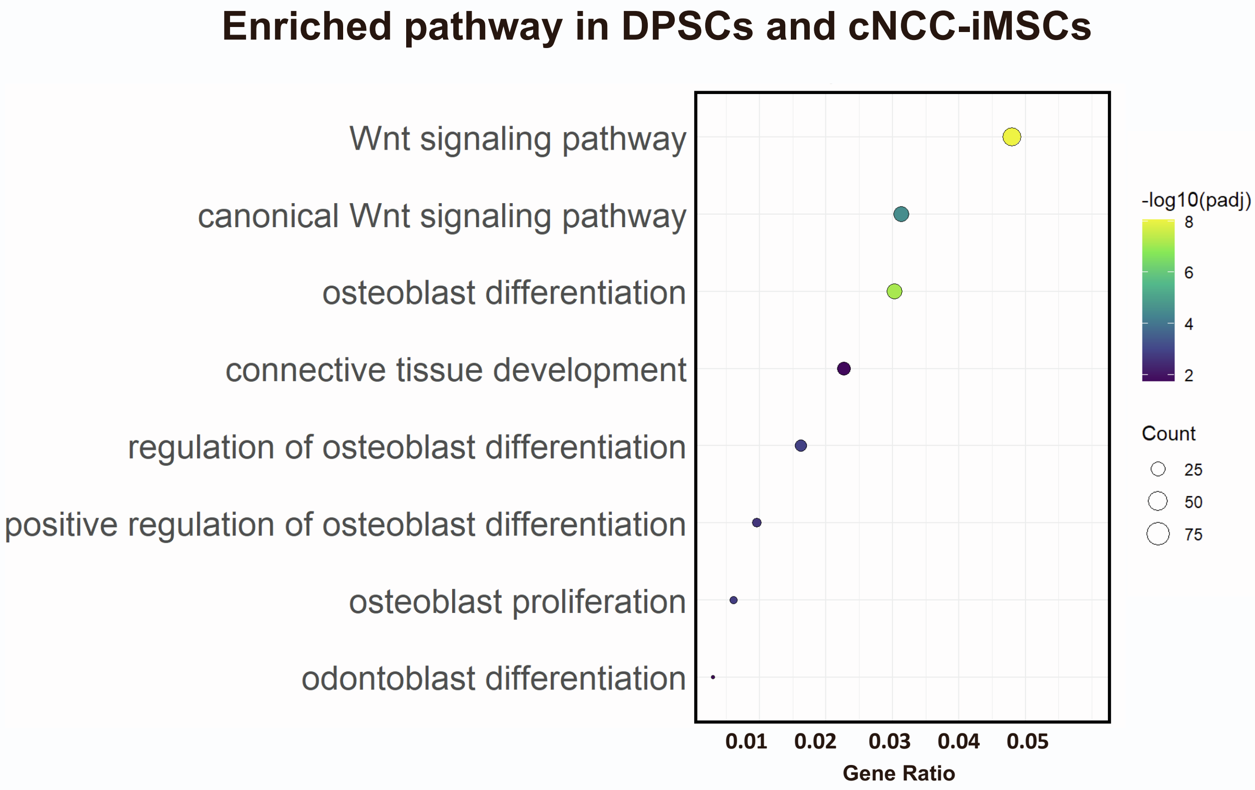

Figure S19. Transcriptomic characteristics of DPSCs and cNCC-iMSCs, Related to Figure 6.
Gene Ontology (GO) terms analysis of the most highly expressed genes in DPSCs and cNCC-iMSCs reveals that both cranial neural crest-derived iMSCs and dental pulp-derived mesenchymal stem cells are enriched in odontogenesis-related pathways. These include the Wnt signaling pathway, osteoblast and odontoblast differentiation.

Table S1. Summary of the morphology, proliferation, and differentiation of iMSCs

Cell type	Morphology	Proliferation	Osteogenesis	Adipogenesis	Chondrogenesis
cNCC-iMSCs	Small, compacted	Moderate	Moderate	Moderate	High
tNCC-iMSCs	Small, compacted	Moderate	Low	Moderate	Moderate
SM-iMSCs	Small, spindle-shaped	High	High	High	High
LPM-iMSCs	Spread, elongated	Low	Low	Moderate	Low
Limb-iMSCs	Small, spindle-shaped	Moderate	High	High	Moderate

iMSCs, induced pluripotent stem cell–derived mesenchymal stem cells; cNCC-iMSCs, cranial neural crest cell–derived iMSCs; tNCC-iMSCs, tissue neural crest cell–derived iMSCs; SM-iMSCs, somite-derived iMSCs; LPM-iMSCs, lateral plate mesoderm–derived iMSCs

Table S3: Summary of all developmental signaling pathway modulators used in this study

Item name	Description	Company	Catalog no.
A83-01	TGFβ inhibitor	Tocris	2939
C59	PORCN inhibitor	Cellagen Technology	C7641-2S
CHIR99021	GSK-3 inhibitor	Tocris	4423
DMH1	BMP inhibitor	Tocris	4126
LDN193189	BMP inhibitor	Stemgent	04-0074
PIK90	PI3K inhibitor	Millipore	528117-5MG
Retinoic acid	Retinoid ligand	Sigma	R2625-50MG
SB-432542	TGFβ inhibitor	Tocris	S1067

Table S4: Proteins/ Growth factors used in this study

Item name	Description	Company	Catalog no.
Activin A	TGFβ ligand	R&D Systems	338-AC-050
BMP4	BMP ligand	R&D Systems	314-BP-050
EGF	EGF ligand	R&D Systems	236-EG
FGF2	FGF ligand	R&D Systems	3718-FB
PDGF-BB	PDGF ligand	R&D Systems	220-BB-200
TGFβ-3	TGFβ-3 ligand	Peprotech Inc.	100-36E

Table S5: Antibodies for flow cytometry in this study

Antibody	Company	Catalog no.	Dilution
CD34 PE	Biolegend	343506	1: 40
CD45 APC	Biolegend	368512	1: 40
CD73 PE	BD Pharmigen	550257	1:100
CD90 APC	Biolegend	328113	1:100
CD105 APC	Invitrogen, eBiosciences™	17-1057-42	1:100
CD271 Alexa fluor 647	BD Pharmigen	560326	1:100
DLL1 APC	R&D Systems	FAB1818A	1:200
CD309 PE	BD Pharmigen	560872	1:100
PDGFRα Alexa fluor 647	BD Pharmigen	562798	1:100

Table S6: Primers for quantitative PCR in this study

Gene name	Forward	Reverse
ACTB	CACCATTGGCAATGAGCGGTTC	AGGTCTTTGCGGATGTCCACGT
ADPOQ	AACATGCCCATTCGCTTTACC	TAGGCAAATAGTACAGCCCA
CEBPB	TTTGTCCAAACCAACCGCAC	CCCCCAAAAGGCTTTGTAACC
CDX2	GGAACCTGTGCGAGTGGAT	TGAAACTCCTTCTCCAGCTCC
COL1A1	GTGCTAAAGGTGCCAATGGT	GTGCTAAAGGTGCCAATGGT
COL1A2	TGCTTGCAGTAACCTTATGCCTA	CAGCAAAGTTCCCACCGAGA
COL2A1	CCTCAAGGCAAAGTTGGTCCT	CTCCCGTCTCACCGTCTTTT
COL10A1	ACGCTGAACGATACCAAACG	GCACACCTGGTTTCCCTACA
FOXF1	AGCAGCCGTATCTGCACCAGAA	CTCCTTTCGGTCACACATGCTG
HAND2	ACATCGCCTACCTCATGGAC	TCCTCCTTCTCCTCCTCCTC
HOXB2	TTCACCAGTACGCTCTGTGC	TTTTCCAGTAGACGCGGCCAAG
HOXB5	AACTCCTTCTCGGGGCGTTAT	CATCCCATTGTAATTGTAGCCGT
HOXB8	TAAGCG GCGAATCGAGGTAT	TGTTTCTCCAGCTCCTCCTG
IDO1	GCCAGCTTCGAGAAAGAGTTG	ATCCCAGAACTAGACGTGCAA
IRX3	AAAAGTTACTCAAGACAGCTT	GGATGAGGAGAGAGCCGATA
ISL1	AGATTATATCAGGTTGTACGGGATCA	ACACAGCGGAAACACTCGAT
MEOX1	GAGATTGCGGTAAACCTGGA	GAACTTGGAGAGGCTGTGGA
MMP13	CATGAGTTCGGCCACTCCTT	CCTGGACCATAGAGAGACTGGA
NKX2-5	CAAGTGTGCGTCTGCCTTT	CAGCTCTTTCTTTTCGGCTCTA
PAX3	AGGAAGGAGGCAGAGGAAAG	CAGCTGTTCTGCTGTGAAGG
PD-L1	TGGCATTTGCTGAACGCATTT	TGCAGCCAGGTCTAATTGTTTT
PHOX2B	CTACCCCGACATCTACACTCG	CTCCTGCTTGCGAAACTTG
PITX1	GACCCAGCCAAGAAGAAGAA	AACTGCTGGCTTGTGAAGTG
PPARG	CCAGAAGCCTGCATTTCTGC	CACGGAGCTGATCCCAAAGT
PPRX1	GAGAAGATCTCGCACGTCGG	TAGCCATGGCGCTGTACG
RUNX2	CAGACCAGCAGCACTCCATA	CAGCGTCAACACCATCATTC
SOX9	AGTACCCGCATCTGCACAAC	ACGAAGGGTCTCTTCTCGCT

SOX10	GAGCTGGACCGCACACCTTGGG	AACGCCCACCTCCTCGGACCTC
TBX5	TACCACCACACCCATCAAC	ACACCAAGACAGGGACAGAC

Table S7: Antibodies for immunostaining in this study

Antibody	Company	Catalog no.	Dilution
COLX	Invitrogen, eBiosciences	14-9771-80	1:200
SOX10	R&D Systems	AF 2864	1:200
p75	Santa Cruz Biotechnology	sc-271708	1:200
TWIST1	Santa Cruz Biotechnology	sc-81417	1:200
